# PLASTID ENVELOPE ION CHANNELS (PEC1/2) link Ca^2+^ and jasmonic acid signaling in plant cells

**DOI:** 10.1073/pnas.2525536123

**Published:** 2026-08-28

**Authors:** Susanne Mühlbauer, Dawid Jaślan, Inês F. Duarte Nunes, Benjamin Brandt, Lena Wutz, Khansa Mekkaoui, Sanja Zenker, Jakob Rehberger, Marlena Rädler, Lorenz Holzner, Constance Tisserant, Carsten Völkner, Andrea Bräutigam, Silke Robatzek, Bettina Hause, Christian Grimm, Hans-Henning Kunz

**Affiliations:** ^a^Plant Biochemistry and Physiology, Faculty of Biology, Ludwig-Maximilians-University Munich, Planegg-Martinsried 82152, Germany; ^b^ Walther Straub Institute of Pharmacology and Toxicology, Faculty of Medicine, Ludwig-Maximilians-University Munich, Munich 80336, Germany; ^c^ Department of Cell and Metabolic Biology, Leibniz Institute of Plant Biochemistry, Halle/Saale 06120, Germany; ^d^ Computational Biology, Faculty of Biology, Bielefeld University, Bielefeld 33615, Germany; ^e^ Department of Computational Biology, Center of Biotechnology, Bielefeld University, Bielefeld 33615, Germany; ^f^Genetics, Faculty of Biology, Ludwig-Maximilians-University Munich, Planegg-Martinsried 82152, Germany; ^g^ Department of Pharmacology, Faculty of Medicine, University of Oxford, Oxford OX1 3QT, United Kingdom; ^h^ Immunology, Infection and Pandemic Research, Fraunhofer Institute for Translational Medicine and Pharmacology, Munich/Frankfurt 80799, Germany

**Keywords:** chloroplast, ion channels, Ca^2+^ signaling, jasmonic acid, stress defense

## Abstract

Plants constantly encounter adverse environmental interactions. One organelle is particularly specialized in stress signaling and phytohormone synthesis: the plastid. Calcium (Ca^2+^), a key second messenger, is known to intersect with cellular phytohormone signaling networks. While cytosolic Ca^2+^ dynamics have been studied extensively, the physiological relevance of stromal Ca^2+^ transients and the identity of channels mediating rapid Ca^2+^ flux into plastids remain largely unexplored. In this study, we provide evidence for PLASTID ENVELOPE ION CHANNELS (PECs) as long-sought mediators of fast-activating cation channel-like currents. We show that *PEC* expression is jasmonic acid (JA)-induced to augment stromal Ca^2+^ transients under stress. Loss of PECs results in decreased JA priming and failure to elicit full defense responses after wounding. In turn, *PEC1* overexpression improves *Botrytis cinerea* tolerance. Our findings link stromal Ca^2+^ signaling with JA-dependent stress responses and position PECs as modulators of plant defense.

## Significance

Plants possess rapid defense responses that help them survive environmental stress. Calcium ions (Ca^2+^) act as universal messengers in these responses. However, their role in plastids, a cell organelle with central function in stress perception, hormone biosynthesis, and adaptation, was unclear. We identified PLASTID ENVELOPE ION CHANNELS (PECs) as mediators of plastid Ca^2+^ signals. *PEC* expression is induced by jasmonic acid (JA) to augment plastid Ca^2+^ signals during reoccurring stress, which sets the plant into a primed state. Loss of PECs disrupts this process, weakening stress-induced JA responses and defense. Conversely, *PEC* overexpression exacerbates plastid Ca^2+^ signals and improves pathogen tolerance. These findings link plastid Ca^2+^ signals with JA-controlled stress responses, and position PECs as mediators of plant defense.

Challenged by abiotic and biotic stress, plants must constantly choose between growth and defense (1–3). This tradeoff is in part regulated by rapid signals such as reactive oxygen species (ROS) and Ca^2+^ transients, which elicit within seconds of stress perception (4, 5). Ca^2+^ acts as both a local and systemic signal, transmitting information within and between leaves (6–8). For instance, herbivory triggers cytosolic Ca^2+^ transients that rapidly activate defense programs through biosynthesis of phytohormones (6, 9–11).

The oxylipin phytohormone jasmonic acid (JA) regulates responses to abiotic stress, nutrient starvation, and development (12, 13). However, it is best known for its role in wound-induced defense (12). JA biosynthesis comprises a genetic feed-forward loop initiated in the plastid, where α-linolenic acid, released from membrane lipids by unresolved lipase(s), is oxygenated by 13-lipoxygenases (LOXs) (14, 15). Subsequent enzymatic processing yields 12-*cis*-oxo-phytodienoic acid (OPDA), which, upon transport into peroxisomes, is converted into JA. Finally, cytosolic JASMONATE RESISTANT 1 (JAR1) conjugates isoleucine to JA to yield bioactive JA-Ile that diffuses into the nucleus. Here, JA-Ile is sensed by CORONATINE-INSENSITIVE 1 (COI1), which degrades JA ZIM domain (JAZ) repressors. This releases MYC2/3/4 transcription factors (TFs) to upregulate JA biosynthesis genes, completing the feed-forward loop (16).

Links between Ca^2+^ and JA signaling have long been proposed: a) Ca^2+^ can promote JA biosynthesis in a MYC-independent fashion via Ca^2+^-dependent disintegration of the nuclear JAV1-JAZ8-WRKY51 suppressor complex (11) and b) some plant lipases and plastid LOXs reportedly require Ca^2+^ for activity (17–20).

Stress-induced cytosolic Ca^2+^ needs to be rapidly redirected into organelles to prevent toxicity (21, 22). Since several compartments possess their own Ca^2+^ signaling pathways, this redistribution can have profound physiological consequences, e.g., modulating Calvin–Benson–Bassham cycle enzymes (23). Chloroplasts, additionally, are capable of actively generating distinct stromal Ca^2+^ transients themselves in response to various stimuli (24–26). However, their physiological relevance remains poorly understood.

The study of mutants defective in cation transport across the plastid inner envelope (IE) may finally provide insights (27). Initially, CHLOROPLAST MITOCHONDRIAL CALCIUM UNIPORTER (cMCU) and BIVALENT CATION TRANSPORTER (BICAT) 2 were identified. However, both are unlikely to mediate rapid Ca^2+^ flux into the stroma of mesophyll chloroplasts, where the initial steps of JA biosynthesis take place, because a) localization of cMCU to chloroplasts is ambiguous and may require further clarification (28, 29), and b) *bicat2* mutants are specifically affected in slow Ca^2+^ kinetics (30). A promising candidate is the FAST-ACTIVATING CHLOROPLAST CATION CHANNEL (FACC), first discovered in pea, which conducts K^+^ > Na^+^ > Ca^2+^ ≥ Mg^2+^ (31, 32). However, the molecular identity of FACC remained unknown.

Recently, two homologs coined PLASTID ENVELOPE ION CHANNELS (PEC1/2) were discovered in pea and *Arabidopsis* envelopes. Heterologous assays done in yeast suggested permeability for K^+^, and *pec1pec2* null mutants exhibit dampened stress-induced stromal Ca^2+^ transients in response to all known stress triggers (33). Because of this highly specific molecular phenotype, we set out to a) test if PECs encode FACC and b) employ *pec1pec2* mutants to gain an understanding of the physiological relevance of rapid stromal Ca^2+^ signals and the role of PECs for plants.

## Results

### PECs Mediate FACC-Like Currents and Facilitate Cytosol-to-Plastid Ca^2+^ Signaling in *Arabidopsis thaliana*.

**Fig. 1.**
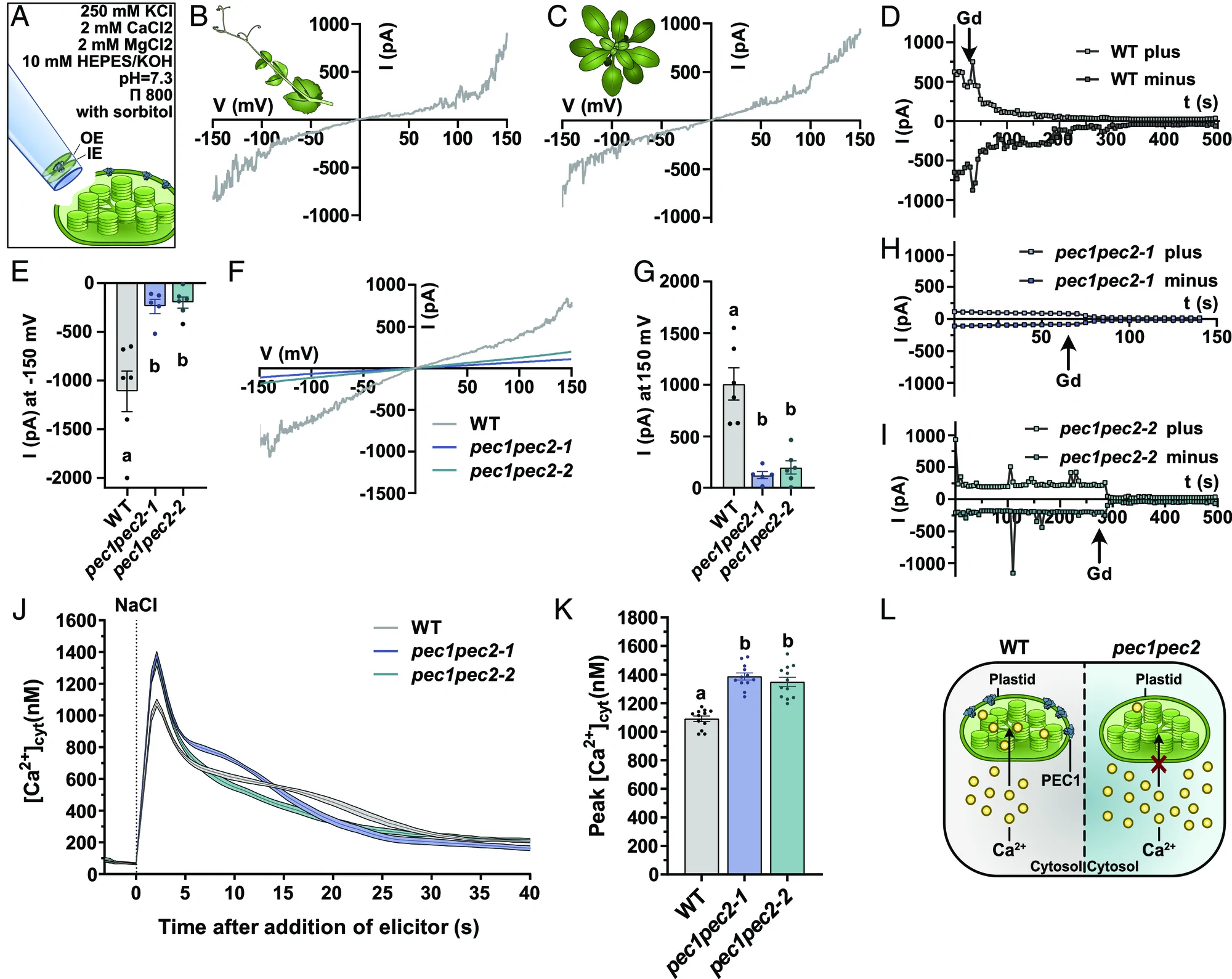
PECs mediate K^+^ and Ca^2+^ flux across the inner chloroplast envelope. (*A*) Schematic representation of the experimental setup for inside-out patch-clamp recordings from isolated chloroplasts. (*B* and *C*) Representative current traces recorded from chloroplasts isolated from *Pisum sativum* (*B*) and *A. thaliana* (*C*) in symmetrical 250 mM KCl solution. (*D*) Representative currents recorded in *Arabidopsis* WT chloroplasts after application of 100 µM GdCl_3_ to the bath solution. (*E*–*G*) Quantification of current traces at −150 mV (*E*) and +150 mV (*G*) from chloroplasts of WT and two independent *pec1pec2* alleles under symmetrical 250 mM KCl conditions. Bars represent mean ± SEM (n ≥ 5). Letters indicate statistically distinct groups (one-way ANOVA followed by Tukey’s post hoc test, *P* < 0.05). (*F*) Representative current traces for WT and *pec1pec2* chloroplasts. (*H* and *I*) Representative traces after application of 100 µM GdCl_3_ to the bath solution of *pec1pec2-1* (*H*) and *pec1pec2-2* (*I*) chloroplasts. (*J*) Cytosolic Ca^2+^ transients measured in WT and *pec1pec2* seedlings following elicitation with 200 mM NaCl. Lines indicate mean, shaded areas indicate SEM (n = 12). (*K*) Quantification of peak cytosolic Ca^2+^ concentrations shown in (*J*). Bars represent mean ± SEM (n = 12). Letters indicate statistical significance (one-way ANOVA with Tukey’s post hoc test, *P* < 0.05). (*L*) Schematic representation showing proposed PEC function: In WT, PECs mediate plastid Ca^2+^ uptake from the cytosol during stress signaling. Loss of PECs traps Ca^2+^ in the cytosol and thus impairs stromal transients.

To determine whether PECs mediate FACC-like currents, we established patch-clamp recordings on isolated *Arabidopsis* chloroplasts. As a reference, we first reproduced previously published electrophysiological measurements from isolated pea chloroplasts (Fig. 1*A*) (31). Intact chloroplasts were identified by the characteristic “halo” [due to the refractive index of undamaged envelope membranes (34)]. Excised inside-out envelope patches in symmetric 250 mM KCl showed current-voltage (I/V) relations closely resembling previous data, with minimal activity at ±50 mV (Fig. 1*B*) (31). Upon stepping the membrane potential to ±150 mV, currents increased significantly.

We next extended this approach to *A. thaliana*, which allowed for the analysis of mutants. FACC-like currents were detected in isolated WT chloroplasts, albeit I/V relations suggested that channel openings in *Arabidopsis* are less clearly voltage-dependent than in pea (Fig. 1*C*). Application of the cation channel blocker Gd^3+^ to the stromal side of the excised patch reduced the open-channel current. Similar to pea FACC, only prolonged incubation (4 to 5 min) led to a complete loss of channel activity (Fig. 1*D* and *SI Appendix*, Fig. S1 *A* and *B*).

To test whether PECs mediate these FACC-like currents in *Arabidopsis*, we performed patch-clamp recordings on isolated chloroplasts from two independent loss-of-function mutants, *pec1-1pec2-1* and *pec1-2pec2-2* (hereafter referred to as *pec1pec2-1* and *pec1pec2-2*, respectively). Recordings were carried out in a double-blind manner. Remarkably, all patches formed with envelope membranes of *pec1pec2* loss-of-function mutants displayed severely reduced currents (Fig. 1 *E* and *G*). Gd^3+^ further reduced these residual currents upon addition, albeit not significantly (Fig. 1 *H* and *I* and *SI Appendix*, Fig. S1 *C* and *D*). To confirm that the FACC-like currents are formed by cations rather than chloride, we next substituted KCl for potassium gluconate in both bath and pipette solutions (*SI Appendix*, Fig. S1*E*). This did not abolish the currents recorded in WT (*SI Appendix*, Fig. S1*F*), indicating that PECs are permeable for cations rather than chloride. Altogether, this strongly suggests that PECs mediate FACC-like currents in *Arabidopsis* chloroplasts.

The pea FACC has been shown to conduct K^+^, Na^+^, Mg^2+^, and Ca^2+^ (31). Concomitantly, *pec1pec2* mutants exhibit strongly diminished stromal Ca^2+^ transients. This suggests that PEC-dependent FACC-like conductance may contribute to Ca^2+^ flux across the IE into chloroplasts, thus linking cytosolic and plastid Ca^2+^ signaling. However, it remained unclear whether these elicitor-induced (NaCl, Mannitol, etc.) stromal Ca^2+^ transients emerge from the cytosol. To investigate this in planta, we introgressed a cytosolic aequorin-based Ca^2+^ sensor [pMAQ2 (35)] into the *pec1pec2* background and quantified cytosolic Ca^2+^ levels. In WT seedlings, we observed large Ca^2+^ transients exceeding 1,000 nM [Ca^2+^]_cyt_ in response to NaCl. This response was stronger in both *pec1pec2* lines, i.e., cytosolic Ca^2+^ transients were significantly elevated compared to WT (Fig. 1 *J* and *K*), which indicates that loss of PECs partially impairs Ca^2+^ flux from the cytosol into plastids, trapping Ca^2+^ in the cytosol. Importantly, this increase in cytosolic Ca^2+^ was mirrored by the reduction of stromal Ca^2+^ transients in *pec1pec2* (*SI Appendix*, Fig. S2 *A*–*C*). This suggests that cytosolic Ca^2+^ transients precede and drive the stromal Ca^2+^ signals during osmotic stress. In WT, PEC-dependent ion flux appears to directly or indirectly connect cytosolic and plastid Ca^2+^ signals by enabling rapid Ca^2+^ entry into the stroma. In *pec1pec2*, this connection is disrupted, which causes cytosolic Ca^2+^ to accumulate, while stromal transients are diminished (Fig. 1*L*).

### PEC Expression Is Controlled by JA-Inducible Transcription Factors.

**Fig. 2.**
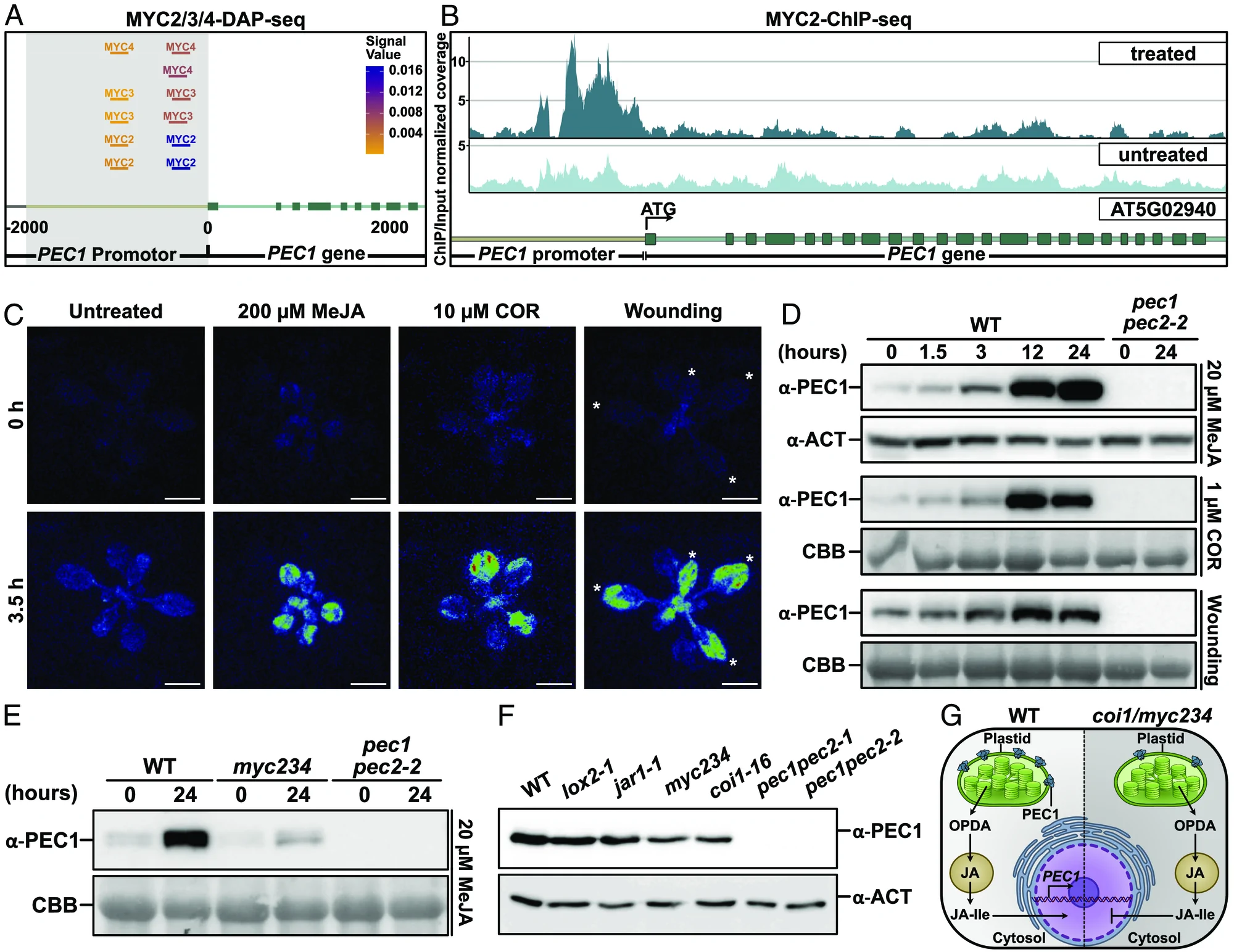
PEC expression is controlled by the MYC branch of the canonical JA signaling pathway. (*A*) DAP-Seq profiles showing MYC2/3/4 binding to the *PEC1* promoter. Binding events are colored from orange to purple by normalized peak height. (*B*) ChIP-Seq showing increased MYC2 binding to the *PEC1* promoter region after water spray treatment compared to the untreated control. Enrichment over input was calculated (bin size = 1 bp), normalized by read count, and plotted over the *PEC1* gene model (Chr5:683630-691192). (*C*) Luciferase activity in *pPEC1::LUC2* reporter lines following treatment with 200 µM MeJA, 10 µM COR, or mechanical wounding. Photon counts were integrated over 3.5 h and images were taken at indicated time points; asterisks mark wounded leaves. Scale bar equals 1 cm. (*D*) Immunoblot analysis of PEC1 protein accumulation in 2-wk-old WT and *pec1pec2-2* at indicated time points following treatment with 20 µM MeJA, 1 µM COR, or wounding. ACTIN or Coomassie Brilliant Blue (CBB) staining of RuBisCO LSU serves as loading control, respectively. (*E*) Immunoblot displaying PEC1 levels in 2-wk-old *myc234* loss-of-function mutants compared to WT and *pec1pec2-2* before and after 24 h of MeJA treatment. CBB staining of RuBisCO functions as loading control. (*F*) Basal PEC1 protein levels in 3-wk-old JA signaling mutants (*lox2-1*, *jar1-1*, *coi1-16*, *myc234*) compared to WT. ACTIN serves as loading control. (*G*) Schematic representation showing that *PEC* expression is induced MYC2/3/4-dependently upon activation of JA signaling.

To gain a deeper understanding of the physiological relevance of PEC function, we investigated the regulatory mechanisms governing *PEC* expression. We screened *PEC1/2* promoter regions (2 kb upstream of the start codon) for conserved TF binding sites using in vitro DNA Affinity Purification Sequencing (DAP-Seq) data (36, 37). This analysis identified binding events involving 74 different TFs in the *PEC1* promoter, and 53 TFs in the *PEC2* promoter (*SI Appendix*, Fig. S3 *A* and *B* and Dataset S1). Gene ontology (GO) enrichment revealed that TFs binding the *PEC1* promoter are linked to stimulus-related hormone responses, whereas those associated with *PEC2* seem more generally involved in development (*SI Appendix*, Fig. S3*C* and Dataset S2). Notably, both promoters contain two conserved G-box motifs (5’-CACGTG-3’), known binding sites for the MYC TF family (38). DAP-Seq confirmed that MYC2, MYC3, and MYC4 bind both G-box motifs in the *PEC1* promoter in vitro, whereas the *PEC2* promoter was only targeted by MYC3 and MYC4 (Fig. 2*A* and *SI Appendix*, Fig. S3*D*). MYC2 is partially redundant with MYC3 and MYC4, and acts as the master coordinator of JA-mediated stress responses (39).

To affirm MYC2 binding to *PEC1* in planta, we further analyzed Chromatin Immunoprecipitation Sequencing (ChIP-Seq) datasets (40, 41). In untreated plants, MYC2 showed little association with the *PEC1* or *PEC2* promoter. However, MYC2 binding to the *PEC1* promoter was strongly enriched in stress-treated plants (Fig. 2*B*), which was also observed for *PEC2* (*SI Appendix*, Fig. S3*E*), albeit to a lower degree, in line with DAP-Seq analysis (*SI Appendix*, Fig. S3*B*). Given that MYC2 is a prominent regulator of JA-mediated responses, this suggests that PEC1 may be the more JA-responsive isoform.

To confirm that JA induces *PEC1* expression, we treated *Arabidopsis* *pPEC1::LUC2* reporter lines with methyl jasmonate (MeJA) or coronatine (COR), a JA-Ile mimic (42), or by wounding and monitored *PEC1*-driven *LUC2* expression (Fig. 2*C*). After 3.5 h, luminescence was considerably higher in all treated plants compared to controls, confirming *PEC1* promoter activation.

This raised the question how JA-dependent *PEC1* promoter regulation translates into transcriptional dynamics during JA signaling. Thus, we reanalyzed transcriptomic data from a 16-h time series following MeJA treatment (43). Similar to established JA marker genes, *PEC1* and *PEC2* transcript abundance increased significantly within 1 to 2 h and gradually declined over the next 14 h. However, *PEC1* expression remained ~2.5 to 20 times higher than *PEC2* at all times (*SI Appendix*, Fig. S4). Broader analysis of JA-responsive genes revealed at least three distinct temporal expression patterns: 1) very early responders, peaking and subsiding within 1 to 2 h (e.g., *OPR3*, *JAR1*) (*SI Appendix*, Fig. S4*A*), 2) rapid and transient responders, peaking within 1 to 4 h and then returning to baseline (e.g., *AOS*, *AOC1*) (*SI Appendix*, Fig. S4*B*), and 3) rapid and sustained responders, which reach peak expression within 1 to 4 h and remain at least 30% elevated throughout the 16 h period (e.g., *LOX2*, *MYC2*) (*SI Appendix*, Fig. S4*C*). Based on this classification, *PEC2* acts as a rapid, transient JA responder, with expression peaking ~2 h posttreatment and returning to baseline by 12 h. *PEC1*, by contrast, shows a rapid and sustained JA-controlled expression pattern, with a broad peak expression between 1 to 4 h posttreatment and no return to baseline. Notably, *PEC1* and *LOX2* share similar expression patterns, with a rapid MeJA-dependent increase followed by sustained expression. This sustained induction pattern, together with its MYC2-dependency and overall higher expression levels, again suggests PEC1 as the more prominent isoform during stress signaling.

To address whether the coinduction of *PEC1* and *PEC2* with JA marker genes reflects potential cofunction, we verified PECs’ temporal- and cell-type-specific expression in *Arabidopsis* (44). This analysis revealed that PECs and selected JA marker genes (e.g., *LOX2*, *AOC2*) have similar diurnal expression patterns (*SI Appendix*, Fig. S4*D*) and are expressed mainly in mesophyll and phloem tissues, confirming that their diurnal and JA-dependent coexpression occurs within the same cell types.

Next up, we monitored PEC1 accumulation over 24 h after treatment with MeJA, COR, or wounding to determine whether the JA-induced expression of *PECs* translates to changes at the protein level. Across all treatments, PEC1 protein levels remained largely unchanged 1.5 h after treatment, increased after 3 h, and accumulated substantially at 12 and 24 h posttreatment (Fig. 2*D* and *SI Appendix*, Fig. S5 *A*–*C*).

Subsequently, we tested the extent to which JA-induced PEC1 accumulation is dependent on the MYC TFs. For this, WT and *myc234* null mutants (39) were treated with MeJA for 24 h. While WT showed a strong accumulation, PEC1 levels in *myc234* remained low (Fig. 2*E* and *SI Appendix*, Fig. S5*D*), which confirms that stress-triggered *PEC1* expression is largely MYC2/3/4-dependent.

Further analysis of basal PEC1 levels in untreated *lox2-1* (45), *jar1-1* (46), *myc234*, and *coi1-16* (47) mutants, all of which harbor defects in key components of the JA signaling pathway, revealed a clear pattern: Basal PEC1 protein accumulation progressively declined with increasing severity of JA signaling disruption (i.e., *myc234* and *coi1-16* exhibit lowest PEC1 levels) (Fig. 2*F* and *SI Appendix*, Fig. S5*E*). This shows that even basal *PEC1* expression, in part, relies on intact JA biosynthesis and signaling. However, residual PEC1 detected in these mutants suggests that basal expression is maintained by additional, JA-independent factors (Fig. 2*G*).

### Stress-Induced PEC Accumulation Augments Stromal Ca^2+^ Signaling.

**Fig. 3.**
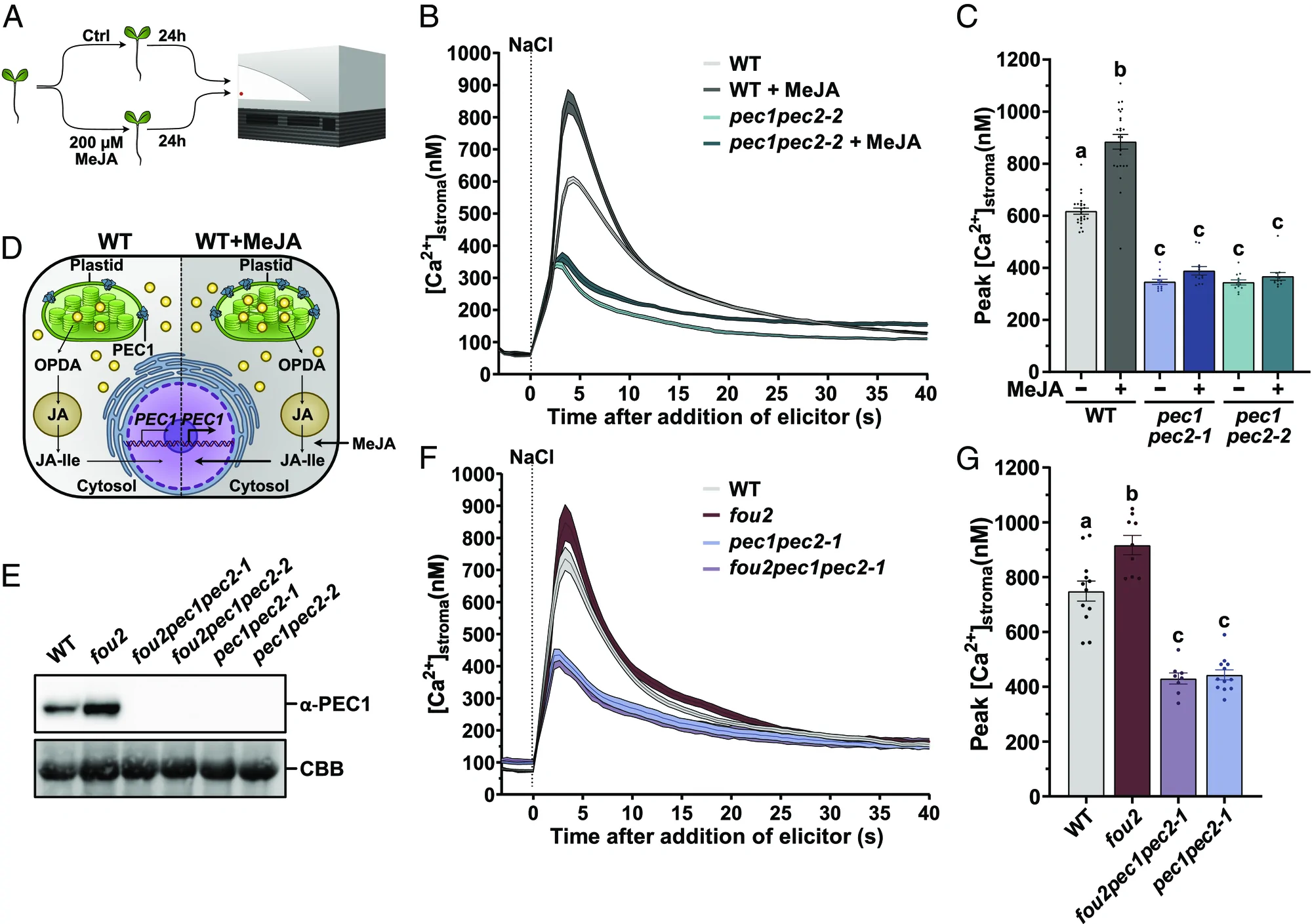
Accumulation of PEC1 augments stromal Ca^2+^ transients JA-dependently. (*A*) Schematic workflow for MeJA treatment and Ca^2+^ quantification. 5-d-old seedlings carrying stromal Aequorin were incubated with 200 µM MeJA or a control solution for 24 h prior to Ca^2+^ measurements. (*B*) Stromal Ca^2+^ transients measured in WT and *pec1pec2-2* seedlings with or without MeJA treatment following elicitation with 200 mM NaCl. Lines indicate mean, shaded areas indicate SEM (n = 24). (*C*) Quantification of peak stromal Ca^2+^ concentrations shown in (*B*). Bars represent mean ± SEM (n = 24). Letters denote statistically distinct groups (one-way ANOVA with Tukey’s post hoc test, *P* < 0.05). (*D*) Schematic model of the proposed mechanism: MeJA treatment induces *PEC1* expression, leading to enhanced plastid Ca^2+^ influx and thus elevated stromal Ca^2+^ transients. (*E*) Immunoblot showing basal PEC1 protein levels in WT, *fou2*, *fou2pec1pec2*, and *pec1pec2* mutants. RuBisCO LSU was stained with CBB as loading control. (*F*) Stromal Ca^2+^ transients in response to NaCl elicitation in WT, *fou2*, *pec1pec2-1*, and *fou2pec1pec2-1* mutants. Lines represent mean, shaded areas indicate SEM (n ≥ 8). (*G*) Quantification of peak stromal Ca^2+^ concentrations shown in (*F*). Bars represent mean ± SEM (n ≥ 8). Letters indicate statistically distinct groups (one-way ANOVA with Tukey’s post hoc test, *P* < 0.05). All data are representative of at least three independent biological replicates.

Given the role of PECs in mediating stromal stress-induced Ca^2+^ transients (33), we investigated whether JA-induced changes in PEC levels alter stromal Ca^2+^ dynamics. Thus, we pretreated *Arabidopsis* WT and *pec1pec2* seedlings expressing plastid-targeted YFP-Aequorin (sAEQ) (33) with or without MeJA (24 h) and subsequently elicited stromal Ca^2+^ signals with NaCl (Fig. 3*A*). WT seedlings pretreated with MeJA exhibited significantly higher stromal Ca^2+^ transients compared to the untreated controls. By contrast, *pec1pec2* mutants displayed consistently dampened Ca^2+^ transients and did not exhibit increased Ca^2+^ levels upon MeJA treatment (Fig. 3 *B* and *C* and *SI Appendix*, Fig. S5 *F*–*H*). This shows that plants can modulate stromal Ca^2+^ levels through JA-controlled PEC expression (Fig. 3*D*).

To further verify our hypothesis, we employed *fou2*, a mutant with constitutively elevated JA levels (48). Since *fou2* accumulated more PEC1 protein already at basal levels (Fig. 3*E* and *SI Appendix*, Fig. S5*I*), we outfitted *fou2* with plastid-targeted YFP-Aequorin and additionally isolated *fou2pec1pec2* triple mutants (*SI Appendix*, Fig. S5*J*). Stromal Ca^2+^ transients in *fou2* were significantly higher compared to untreated WT and largely resembled those of MeJA-treated WT. By contrast, *fou2pec1pec2* triple mutants, like *pec1pec2*, exhibited strongly diminished stromal Ca^2+^ transients in response to NaCl (Fig. 3 *F* and *G*). This confirms that JA signaling tunes stress-induced stromal Ca^2+^ signals mainly by controlling PEC expression (Fig. 3*D*).

### Loss of PECs Impairs Defense Gene Expression and JA Accumulation.

**Fig. 4.**
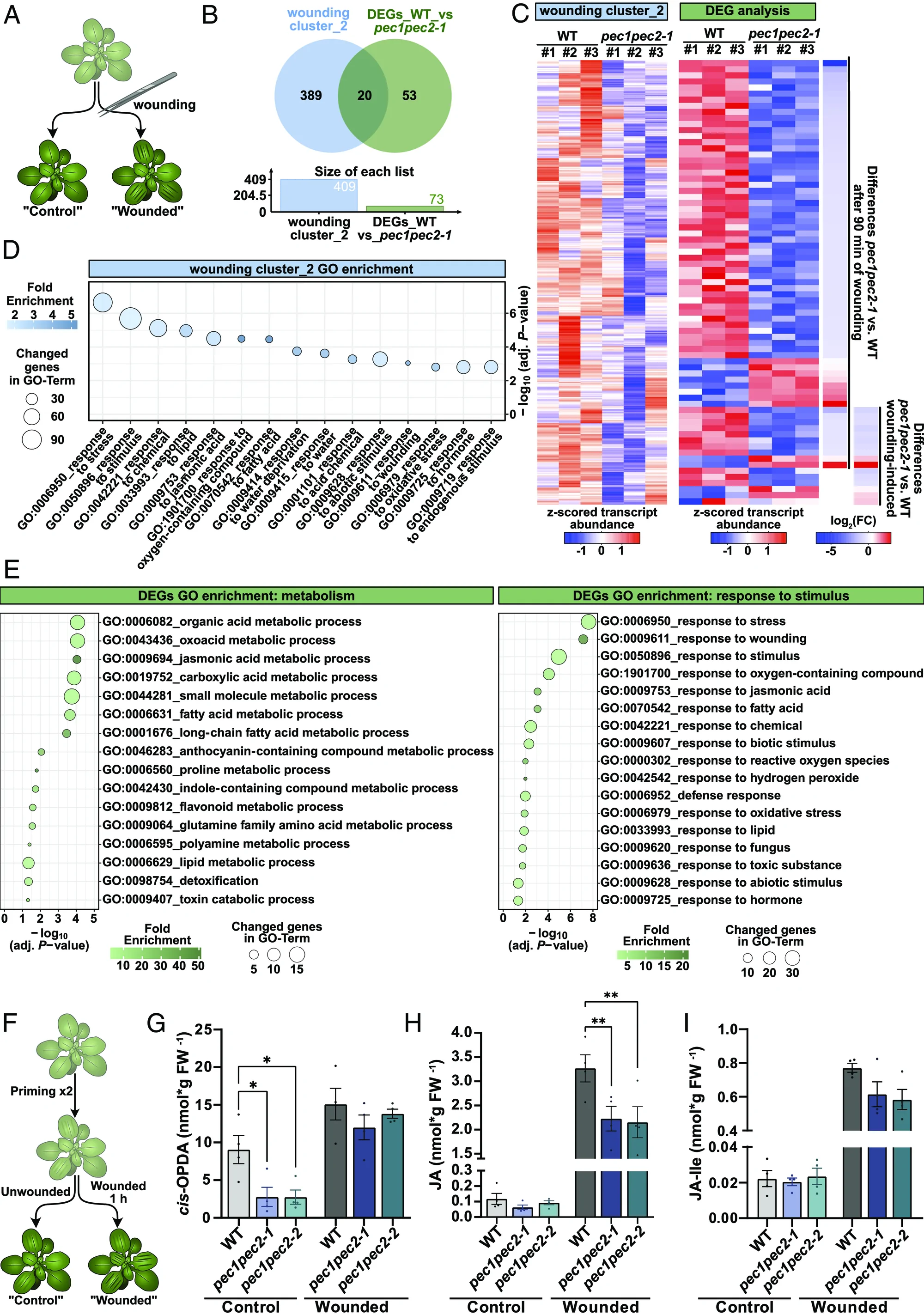
Loss of PECs attenuates wounding-induced transcriptional and hormonal responses. (*A*) Schematic overview of the experimental workflow for short-term transcriptomic analysis. 3-wk-old WT and *pec1pec2-1* mutants were wounded and harvested after 90 min. (*B*) Venn diagram showing the overlap between genes in k-means cluster_2 (*SI Appendix*, Fig. S6*A*) and differentially expressed genes (FDR < 0.05) in *pec1pec2-1* and WT, and after wounding (*pec1pec2* DEGs). (*C*) Heatmaps of expression profiles (*z*-scored transcript abundance) for genes in k-means cluster_2 (*Left*) and *pec1pec2* DEGs (*Middle*). For *pec1pec2* DEGs, corresponding log_2_FCs are shown on the *Right*. (*D*) Selected enriched GO-terms of genes in k-means cluster_2 (Benjamini–Hochberg FDP < 0.05). Circle size corresponds to the number of cluster_2 genes in the GO term, and coloring depicts fold enrichment. (*E*) Selected enriched GO terms of *pec1pec2* DEGs. Circle size corresponds to the number of DEGs in the GO term, and coloring depicts fold enrichment. (*F*) Experimental workflow of hormone quantifications. WT and *pec1pec2* seedlings were primed twice by wounding, then subjected to a final wounding or left untreated (Control). Samples were harvested 60 min after the final treatment. (*G*–*I*) Quantification of *cis*-OPDA (*G*), JA (*H*), and JA-Ile (*I*) in primed WT and *pec1pec2* after wounding or at control conditions (n = 4). Bars represent mean ± SEM. Statistical significance was determined within each treatment by two-way ANOVA with Šidák correction, *P < 0.05, **P < 0.01.

For an overview of the downstream effects of altered stromal Ca^2+^ dynamics, we performed RNA-Seq on *pec1pec2-1* and WT plants under control conditions and 90 min after wounding (Fig. 4*A*). Initially, gene expression patterns were explored by k-means clustering (k = 6) of the top 2,000 most variable genes 90 min after wounding (*SI Appendix*, Fig. S6*A*). Cluster_2 (409 genes, Dataset S3) displayed the most uniform expression changes, and heatmap visualization revealed a consistent trend toward lower expression of cluster_2 genes in *pec1pec2-1* (Fig. 4 *B* and *C*). Interestingly, GO term enrichment of genes in cluster_2 indicated significant overrepresentation of genes involved in stress-related processes i.e., response to water deprivation, wounding, cold, and JA (Fig. 4*D* and Dataset S4). Differentially expressed genes (DEGs) were identified by a statistical analysis across the entire dataset. This allowed us to identify differences in steady state transcriptional abundances between untreated and wounded WT and *pec1pec2-1* plants and to assess how transcriptional wounding responses differ between genotypes. A heatmap of the most diverse genes and Principal Component Analysis (PCA) revealed that the overall transcriptional wounding response remains largely intact in *pec1pec2-1*, and, in line with the 16-h time series, revealed ~6 to 7 times higher expression of *PEC1* compared to *PEC2* in WT (*SI Appendix*, Figs. S6*B* and S7 *A* and *B*). However, we identified 73 DEGs in *pec1pec2-1* compared to WT in response to wounding, out of which only 20 were shared with cluster_2 (Fig. 4*B*). 60 DEGs were downregulated and only 13 upregulated in *pec1pec2-1* vs. WT (Fig. 4*C* and Dataset S5). These genes were enriched for two major functional groups: “metabolism” (e.g., JA and fatty acid metabolism) and “response to stimulus” (e.g., wounding, oxygen-containing compounds, hormone, and JA) (Fig. 4*E* and Dataset S6). Intriguingly, the downregulated genes included JA pathway components such as *LOX3* and *JAR1* (*SI Appendix*, Fig. S7*C*). This indicates that while the global response to wounding in *pec1pec2* remains largely intact, loss of PECs attenuates expression of a subset of JA-related, wound-induced genes, which leaves the plant unable to elicit the full defense response.

As *PEC1* expression is rapidly induced by JA, our transcriptomic analysis mostly focused on its effects on the early wound response. However, *PEC1* expression, like that of *LOX2*, remains elevated long after JA induction (*SI Appendix*, Fig. S4*C*). Thus, we decided to further explore its role in the sustained phase of the defense response by examining PEC1 and LOX2 protein levels over 96 h after wounding in WT and *pec1pec2*. In WT, LOX2 and PEC1 levels strongly increased between 24 to 48 h and remained elevated over 96 h, consistent with their prolonged expression after MeJA treatment. Strikingly, in *pec1pec2*, LOX2 protein levels did not increase notably at any point during the 96-h period beyond basal level (*SI Appendix*, Fig. S7 *D* and *E*). Thus, PECs seem crucial for sustained LOX2 protein accumulation after stress.

Since this implies that loss of PECs is most disruptive days after wounding, we examined hormone levels during sustained stress. To this end, we primed WT and *pec1pec2* mutants by wounding twice during development followed by a final wounding or mock treatment (=unwounded) 60 min before sampling, and quantified *cis*-OPDA, JA, and JA-Ile (Fig. 4*F*). In primed but unwounded plants, OPDA was reduced to ~30% of WT levels in both *pec1pec2* mutants (Fig. 4*G*). This trend persisted after wounding but was no longer significant. Reduced OPDA accumulation during priming also affected JA levels: While JA was equally low in primed but unwounded plants, *pec1pec2* accumulated only ~66% of WT JA levels after a final wounding (Fig. 4*H*). JA-Ile followed JA levels by trend, reaching only ~75% of WT levels in *pec1pec2* after a final wounding (Fig. 4*I*).

In unprimed plants, hormone levels showed a similar pattern that was generally less pronounced (*SI* *Appendix*, Fig. S7 *F*–*I*). In conjunction with prolonged *PEC1* expression, this indicates that PECs may play a more prominent role in sustained defense rather than the immediate wounding response.

### *PEC1* Overexpression Exacerbates Stress-Induced Stromal Ca^2+^ Transients, Augments the JA-Dependent Priming Response, and Increases Resistance Against *Botrytis cinerea*.

**Fig. 5.**
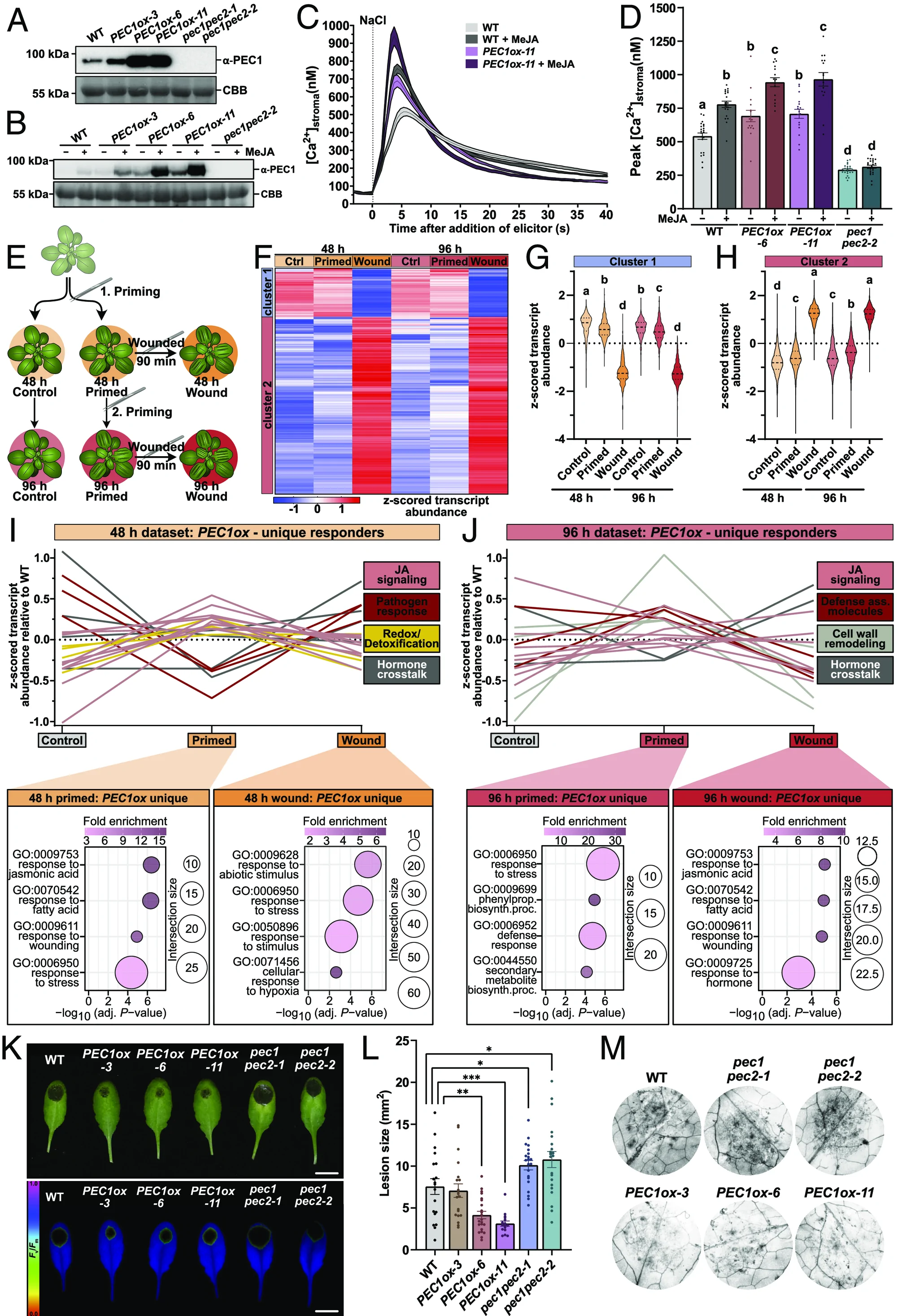
*PEC1* overexpression enhances stromal Ca^2+^ transients, the JA-dependent priming response, and resistance to *B. cinerea.* (*A*) Immunoblot showing PEC1 protein levels in WT, *pec1pec2*, and three independent *PEC1* overexpression lines (*PEC1ox-3/-6/-11*). Coomassie Brilliant Blue (CBB) staining of RuBisCO LSU serves as loading control. (*B*) Immunoblot confirming JA-responsiveness of the *pPEC1::PEC1* construct. Two-wk-old plants were treated with 20 µM MeJA for 24 h. CBB staining of RuBisCO indicates equal loading. (*C*) Stromal Ca^2+^ transients in response to 200 mM NaCl in WT, *pec1pec2*, and *PEC1ox-11*. Shaded areas indicate SEM (n ≥ 16). (*D*) Quantification of peak Ca^2+^ levels from (*C*) and *pec1pec2-2* and *PEC1ox-6* (*SI Appendix*, Fig. S9 *B* and *C*). Data represent mean ± SEM (n ≥ 16). Letters indicate statistical significance (one-way ANOVA with Tukey’s test, *P* < 0.05). (*E*) Schematic overview of the experimental workflow for long-term transcriptomic analysis. Plants were either left untreated as time-matched controls or subjected to priming. WT, *PEC1ox-6*, and *PEC1ox-11* mutants were primed by wounding and harvested 48 h or 96 h after priming. For 96 h samples, plants were subjected to a second priming at 48 h. In parallel, primed plants were wounded at the respective time points and harvested 90 min later to assess the acute wounding responses. (*F*) Hierarchically clustered heatmaps of the *z*-scored transcript abundances (z-scores of normalized read counts) of genes differentially expressed in WT upon wounding (Wound vs. Primed) at 48 and 96 h (*SI Appendix*, Fig. S10*A*) at control, Primed, and Wound conditions. (*G* and *H*) Violin plots showing the z-scored transcript abundances of genes in DEG Cluster 1 (*G*) and Cluster 2 (*H*) identified from (*F*) during control conditions, priming and acute wounding. Letters indicate statistically distinct groups (Kruskal-Wallis test followed by Dunn’s post hoc test, *P* < 0.05). Dashed lines indicate the median, while the dotted lines denote the upper and lower quartiles. (*I*) Line plots showing z-scored transcript abundances in *PEC1ox-6* relative to WT at 48 h. The selected DEGs are unique in *PEC1ox* at 48 h, either in Primed (Primed vs. Control) or Wound (Wound vs. Primed) contrasts (*SI Appendix*, Fig. S11 *A* and *B*). Displayed *PEC1ox* unique DEGs are color-coded by functional category: JA signaling, pathogen response, redox/detoxification, and hormone crosstalk. Bubble plots below show the top four significantly enriched GO terms (g:SCS-corrected, *P* < 0.05) among all *PEC1ox* unique DEGs at 48 h Primed and Wound contrasts. Bubble size corresponds to the intersection size (number of DEGs annotated to the GO term), and coloring depicts fold enrichment. (*J*) Line plots showing z-scored transcript abundances in *PEC1ox-6* relative to WT at 96 h. The selected DEGs are unique in *PEC1ox* at 96 h, either in Primed (Primed vs. Control) or Wound (Wound vs. Primed) contrasts (*SI Appendix*, Fig. S11 *C* and *D*). Displayed *PEC1ox* unique DEGs are color-coded by functional category: JA signaling, defense associated molecules, cell wall remodeling, and hormone crosstalk. Bubble plots below show the top four significantly enriched GO terms (g:SCS-corrected, *P* < 0.05) among all *PEC1ox* unique DEGs at 96 h Primed and Wound contrasts. Bubble size corresponds to the intersection size (number of DEGs annotated to the GO term), and coloring depicts fold enrichment. (*K*) RGB and false color panel showing *F*_v_/*F*_m_ of representative lesions in adult leaves of 5-wk-old WT, *pec1pec2*, and *PEC1ox-3/-6/-11* 3 d after infection with *B. cinerea*. (*L*) Quantification of the lesion size from (*K*). Bars represent mean ± SEM (n ≥ 16). Statistical significance was assessed by unpaired two-tailed *t* test against WT (*P < 0.05; **P < 0.01; ***P < 0.001; *P* < 0.0001). (*M*) Trypan Blue staining of infected leaf discs 2 d postinfection to analyze primary lesion formation. Images are representative of six leaves per genotype.

Since loss of PECs attenuates stromal Ca^2+^ transients and overall stress response, we wondered whether *PEC1* overexpression would similarly alter plastid Ca^2+^ signals and potentially benefit defense. For this, we generated *pec1pec2* mutants stably expressing the *PEC1* genomic locus under its native promoter but fused to the *HSP18.2* terminator (49), known to stabilize mRNA (50). Three independent overexpression lines designated *PEC1ox-3*, *PEC1ox-6*, and *PEC1ox-11* were isolated, all accumulating more PEC1 protein than WT to varying degrees. *PEC1ox-3* showed moderately increased PEC1 levels, while *PEC1ox-6* and *PEC1ox-11* were strong overexpressors (Fig. 5*A*). We verified that the cloned *PEC1* promoter region remained JA-responsive, as MeJA treatment further increased PEC1 protein levels substantially in all overexpression lines to a similar extent as in WT (Fig. 5*B* and *SI* *Appendix*, Fig. S8*A*).

Phenotypic appearance, photosynthetic performance, and chlorophyll content remained largely unaffected in *PEC1* overexpressors. However, all *PEC1ox* lines were smaller and accumulated less fresh weight than WT or *pec1pec2*, which may reflect a growth penalty, typical for mutants with elevated JA levels (51) (*SI Appendix*, Fig. S8 *B*–*I*). To ensure that this phenotype does not stem from general alterations in the plastid ionome caused by *PEC1* overexpression, we analyzed the total leaf and chloroplast ionome (52). Metal ion content of whole rosettes as well as isolated chloroplasts was unchanged in both *pec1pec2* and *PEC1ox* lines (*SI Appendix*, Fig. S8 *J*–*M* and Dataset S7). This further indicates that PECs are not involved in general plastid cation loading but fulfill a highly specialized role, likely conducting only under specific physiological conditions i.e., during stress-induced Ca^2+^ signaling.

Thus, we hypothesized that *PEC1* overexpression may enhance stress-induced stromal Ca^2+^ transients, and with this, plant defense. To test this, we outfitted *PEC1ox-6* and *PEC1ox-11* with plastid-targeted YFP-Aequorin (*SI Appendix*, Fig. S9*A*). Already at baseline, *PEC1ox-6* and *PEC1ox-11* exhibited significantly enhanced NaCl-induced stromal Ca^2+^ transients comparable to MeJA-treated WT, and MeJA treatment exacerbated stromal Ca^2+^ transients even further (Fig. 5 *C* and *D* and *SI* *Appendix*, Fig. S9 *B* and *C*).

Enhanced stromal Ca^2+^ transients may alter JA responses. Therefore, we assessed *PEC1ox* sensitivity to MeJA-induced primary root growth inhibition (46). *PEC1ox* exhibited shorter roots than WT under control conditions (*SI Appendix*, Fig. S9 *D* and *E*), and MeJA further exacerbated this effect, mirroring the *fou2* mutant (*SI Appendix*, Fig. S9 *F* and *G*). Conversely, *pec1pec2* roots developed similarly to WT under control conditions (*SI Appendix*, Fig. S7 *D* and *E*), but, surprisingly, grew significantly longer on MeJA plates, resembling the MeJA-insensitive *myc234* mutants (39) in root length, but not overall appearance (*SI Appendix*, Fig. S9 *F* and *G*). This indicates that changes in *PEC* expression affect JA sensitivity.

Next, we determined whether *PEC1* overexpression alters defense priming i.e., whether sustained wounding shifts the basal and short-term wound-induced transcriptomic profile over a medium (48 h) or prolonged (96 h) time frame, mirroring the JA quantifications (Fig. 4 *F*–*I*). To that end, we performed RNA-Seq on unwounded and wounding-primed WT, *PEC1ox-6*, and *PEC1ox-11* plants. Tissue was collected at 48 h after an initial wounding to capture the primed state, or 90 min after a second wounding applied at that 48 h point to capture the acute transcriptional response. For an extended analysis, plants were wounded a second time at 48 h and then wounded again at 96 h, generating a double-primed state prior to sampling (Fig. 5*E*).

To assess whether transcriptomes are changed during defense priming in WT plants, we selected wound-responsive genes differentially expressed at both 48 and 96 h (*SI Appendix*, Fig. S10*A* and Dataset S8). Hierarchical clustering of transcript abundances separated genes into two major clusters: genes mildly yet significantly repressed during priming and strongly downregulated after wounding (Cluster 1), and genes slightly elevated during priming and further induced upon wounding (Cluster 2; Fig. 5 *F*–*H*). Notably, both clusters showed a significantly stronger priming response (Primed) at 96 h after a second priming, which indicates that repeated stress progressively reinforces plant defense on a transcriptional level. However, the acute short-term wounding response (Wound) was comparable between plants primed for 48 and 96 h, suggesting that the depth of the primed state does not further amplify the immediate transcriptional response to wounding. GO term enrichment revealed distinct differences between both clusters: Cluster 1 was enriched for genes associated with circadian clock and light-responsive processes (*SI Appendix*, Fig. S10*B* and Dataset S9). By contrast, Cluster 2 showed significant overrepresentation of genes involved in stress-related processes, i.e., response to stress, wounding, and JA (*SI Appendix*, Fig. S10*C* and Dataset S9), indicating that the upregulated component of the priming response is in part JA-dependent.

To test whether the defense priming relies on PECs, we compared the transcriptomic responses between *PEC1ox* lines and WT. We focused on genes uniquely responsive in both *PEC1ox-6* and *PEC1ox-11* relative to WT, applying a moderate fold-change threshold for the primed state (|log_2_FC| > 0.5) and a more stringent threshold for acute wounding (|log_2_FC| > 1), reflecting the stronger transcriptional changes induced by direct wounding (*SI Appendix*, Fig. S11 *A*–*D* and Dataset S8).

At 48 h priming, GO term enrichment of *PEC1ox* unique genes identified JA signaling related genes as the most prominent category, which indicates that *PEC1* overexpression modulates the JA-dependent primed state (Fig. 5*I* and Dataset S9). To visualize transcriptional patterns within *PEC1ox* uniquely induced genes, we plotted z-scored transcript abundance relative to WT for selected genes associated with JA signaling, redox/detoxification, pathogen response, and hormone crosstalk, using *PEC1ox-6* as the representative *PEC1ox* line. In line with the GO term enrichment, at 48 h Primed, *PEC1ox* showed a stronger induction of core JA signaling genes together with redox/detoxification markers, while selected pathogen response and hormone crosstalk genes were more repressed (Fig. 5*I* and *SI Appendix*, Fig. S11*E*). Upon rewounding (48 h Wound), JA- and redox-related genes in WT reached expression comparable to *PEC1ox*, whereas pathogen response and hormone crosstalk genes were more strongly induced in the overexpression line. This indicates that *PEC1* overexpression shifts specifically the primed state toward JA-dependent processes, while during acute wounding, it mainly results in enhanced pathogen response and hormone crosstalk.

At 96 h Primed, *PEC1ox*-specific differences in core JA-associated genes became less pronounced (Fig. 5*J*). Instead, the *PEC1ox* unique priming response after 96 h was characterized by enhanced expression of genes linked with defense-associated molecules and cell wall remodeling (Fig. 5*J* and *SI Appendix*, Fig. S11*F*). This was also reflected in the GO term enrichment, which shifted toward broader defense and biosynthetic metabolism (Dataset S9). Upon acute wounding (96 h Wound), *PEC1ox* again showed a stronger induction of some JA and hormone crosstalk related genes, indicating that *PEC1* overexpression continues to affect the transcriptional response, and JA dependent gene expression, even after prolonged priming (Fig. 5*J*). Overall, the transcriptomic responses during defense priming were complex, with aspects of the response varying across time points and wounding regimes. Nevertheless, one feature emerged consistently: JA-related processes were disproportionately represented among PEC1-dependent priming responses, both in terms of GO term enrichment and individual gene expression patterns.

Since JA signaling is a key defense pathway against necrotrophic pathogens (12), we finally tested whether these changes in JA-related processes are reflected in resistance against *B. cinerea*. When infected with *B. cinerea*, strong overexpressors *PEC1ox-6* and *PEC1ox-11* developed significantly smaller lesions and accumulated less fungal biomass than WT, while moderate expressor *PEC1ox-3* behaved statistically indistinguishable from WT. By contrast, *pec1pec2* displayed in most replicates significantly larger lesions compared to WT, as well as stronger primary lesions (Fig. 5 *K*–*M* and *SI Appendix*, Fig. S12*A*).

Together, these results establish a functional link between PEC-mediated regulation of stromal Ca^2+^ signaling and the modulation of downstream JA-mediated physiological defense responses.

## Discussion

The ability of plastids to integrate environmental signals is central to their role in plant stress adaptation as they host key biosynthesis and regulatory steps for stress-related hormones such as JA, SA, and ABA (53). While Ca^2+^ signaling reportedly intersects with all three (54), most studies have focused on their links to cytosolic Ca^2+^ dynamics. By contrast, the physiological significance of stress-induced stromal Ca^2+^ transients, and plastid Ca^2+^ influx mechanisms remain largely unknown (27). Electrophysiological studies on pea chloroplasts identified the FACC with high Ca^2+^ permeability in the inner envelope (31). However, its molecular identity remained elusive. In *Arabidopsis*, we observed a FACC-like, highly conductive envelope channel, which was absent in *pec1pec2* mutants that exhibited strongly diminished envelope currents and ~80% reduced K^+^ conductance (Fig. 1 *E* and *G*). Residual currents indicate the presence of other K^+^ or Cl^−^ permeable channels with lower conductivity than PEC1/2 [e.g., MSL2/3 (55, 56)].

Channel gating for PECs revealed weaker voltage dependence than for pea FACC, which may relate to physiological differences, the presence of distinct isoforms, or species-specific regulation. Importantly, PEC-mediated currents share key characteristics with pea FACC, including permeability for K^+^ (33), high total conductance, rapid activation, and Gd^3+^ sensitivity. These properties position PECs as best candidates for the FACC to date.

FACC has confirmed Ca^2+^ permeability and exhibits high open probability at physiological cytosolic Ca^2+^ concentrations. Concomitantly, loss of PECs diminishes stress-induced stromal Ca^2+^ transients (33) while simultaneously trapping Ca^2+^, normally directed toward plastids, in the cytosol (Fig. 1 *J* and *K*). Therefore, PECs provide a mechanism to manipulate stress-induced cellular and especially chloroplast Ca^2+^ signaling and enable investigation of the physiological function of stromal Ca^2+^ transients.

Promoter analysis revealed that PECs represent direct targets of MYC2/3/4 TFs (Fig. 2 *A* and *B*), master coordinators of JA signaling (39). This aligns with recent research adding *PEC1* as a MYC2 core target (57). Time-resolved transcriptomics of MeJA treatments show that *PEC1* is JA-inducible and follows a sustained induction profile, peaking at around 1 to 3 h posttreatment but subsiding slowly thereafter over 16 h (*SI Appendix*, Fig. S4). This sustained expression pattern contrasts with early canonical JA biosynthesis genes such as *JAR1* and *OPR3*, which peak rapidly and return to baseline within 4 h, and instead parallels that of *LOX2* and *MYC2*.

Like LOX2, which is essential for sustaining JA biosynthesis beyond the initial burst 90 min postwounding (45), MYC2 mediates not only immediate wounding responses but also contributes to long-term immune memory (58). PEC1 protein levels increase robustly following MeJA treatment, wounding, and coronatine exposure (Fig. 2 *C* and *D*), and, like LOX2, remain elevated for more than 96 h after wounding (*SI Appendix*, Fig. S7*D*). This indicates that PECs may function as part of a sustained plastid amplification circuit. Our data show that JA-dependent control of *PEC* expression actively augments stromal Ca^2+^ transients: Stress exposure leads to higher PEC protein levels and thus amplified stromal Ca^2+^ flux (Fig. 3 *B* and *C*). This mechanism may represent a physiological strategy linking rapid Ca^2+^ transients, which rise seconds after stress perception, to long-distance sustained hormone signaling (59).

Plant lipoxygenases e.g., LOX2, which catalyzes the first committed JA biosynthesis step in the stroma, are Ca^2+^-dependent, a requirement also conserved in animal LOXs, to associate LOX with membrane lipids (17, 18, 60, 61). Ca^2+^-dependent regulation has also been reported for some plant lipases (20). Coexpression and highly similar temporal behavior of *PEC1* and *LOX2* suggest a functional interplay in which JA-dependent upregulation of PECs ensures that stromal Ca^2+^ supply meets the demand for plastid oxylipin biosynthesis during priming and sustained defense. This may facilitate the activation of Ca^2+^-responsive enzymes, e.g., LOX2, and potentially influence candidate lipases for the release of α-linolenic acid during the initiation of JA signaling, such as DAD1-LIKE LIPASES (DALLs) (19) or other as-yet-unidentified lipases. However, during early defense, PEC-dependent modulation of stromal Ca^2+^ transients appears supportive rather than essential to elicit the full stress response (Fig. 4 *B*–*E*).

This hypothesis is supported by a significant reduction of plant hormone levels only in primed *pec1pec2* mutant plants (~30% OPDA, ~66% JA) compared to WT (Fig. 4 *G* and *H*), coinciding with increased susceptibility to *B. cinerea* (Fig. 5 *K*–*M*). This suggests that PECs support plastid-derived oxylipin precursor biosynthesis during prolonged or repeated stress, ensuring a stronger and faster hormonal response. Modulating PEC protein levels shapes stromal Ca^2+^ signaling, and, by extension, JA-dependent defense. Consequently, *PEC1* overexpression exacerbates stromal Ca^2+^ transients, both JA-mediated priming and broader wounding-induced defense responses on a transcriptional level, and thus ultimately enhances resistance to *B. cinerea* without disturbing chloroplast ion composition or photosynthesis (Fig. 5 and *SI Appendix*, Figs. S8–S12). Altogether, this suggests that PECs do not mediate general plastid cation loading but are tightly regulated and fulfill highly specialized roles in modulating stress signaling. While *PEC1* overexpressors exhibit modest growth reductions (*SI Appendix*, Fig. S8*C*), hallmarks of enhanced JA signaling (51), these effects were mild compared to known JA-overproducing mutants such as *fou2* and *cev1* (48, 62). Thus, tuning PEC1 levels may offer a well-dosed avenue to modulate defense while minimizing growth tradeoffs.

**Fig. 6.**
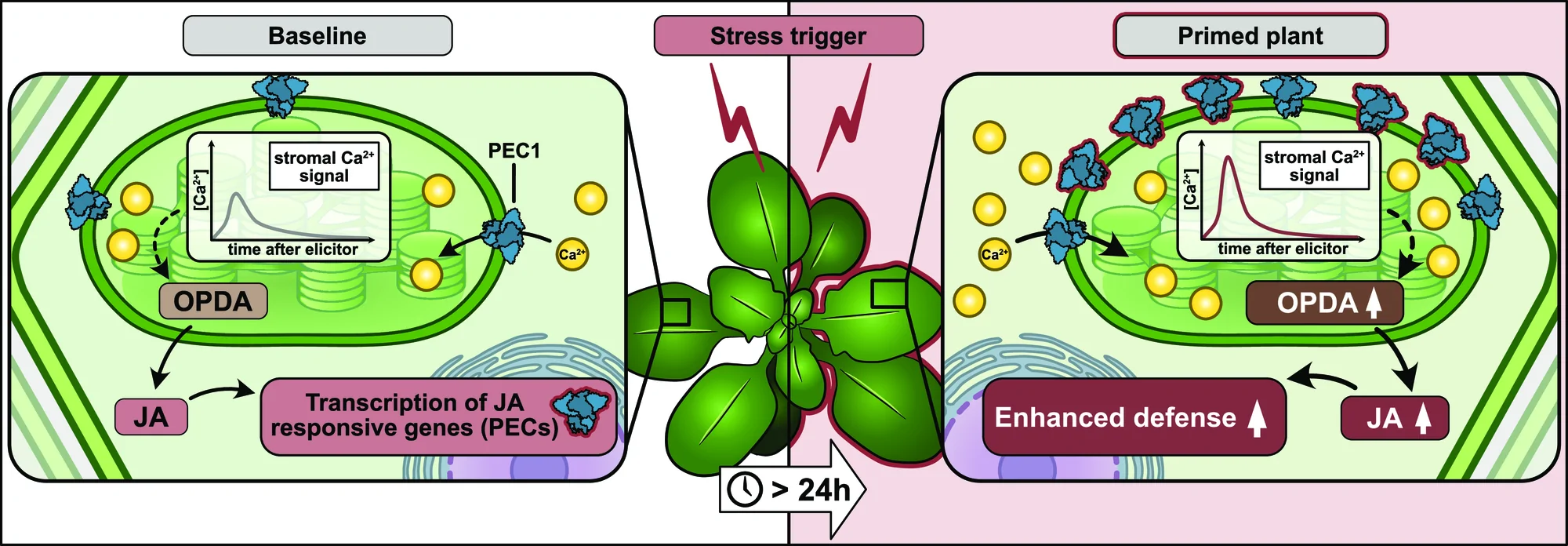
PECs are modulators of the plant defense response. Working model: Under basal conditions, PEC1 levels are low, directly or indirectly limiting plastid Ca^2+^ influx. Upon priming or stress, *PEC1* expression is induced controlled by JA signaling. ≥24 h after priming, elevated PEC1 protein levels enhance stromal Ca^2+^ transients, which allows for the accumulation of higher levels of *cis*-OPDA, which translates into higher JA and JA-Ile levels upon trigger. This ultimately likely enables a faster and stronger defense response. Note, the plastid outer envelope was omitted for simplicity.

In summary, we propose a model in which PECs and the stromal Ca^2+^ transients they mediate act as modulators within plant defense (Fig. 6). Our data show that PECs are not essential for early Ca^2+^-dependent defense responses, likely due to Ca^2+^ saturation at this stage and/or compensatory mechanisms, e.g., unknown ion channels that maintain sufficient relay of cytosolic and stromal Ca^2+^ signals in *pec1pec2*. However, prolonged stress and concomitant JA-dependent upregulation of *PEC* expression set the plant cells into a primed state. In this state, increased PEC-dependent Ca^2+^ flux promotes strong and fast activation of lipases or coexpressed lipoxygenases e.g., LOX2, ensuring an enhanced JA response upon recurring stress (Fig. 6). Importantly, PECs do not only affect stromal Ca^2+^ transients: Through altering compartmentalization of stress-induced Ca^2+^ signals between cytosol and plastids, changes in PEC protein levels may further affect downstream Ca^2+^ decoding in the cytosol. This likely contributes to the phenotypes observed here, e.g., reduced MeJA sensitivity in *pec1pec2* mutants. Altogether, this study establishes PECs as modulators that link plastid Ca^2+^ dynamics and cellular compartmentalization to JA-dependent stress defense responses and provides a genetic tool to dissect how compartment-specific Ca^2+^ signatures shape hormonal signaling pathways in plants.

## Materials and Methods

All mutants isolated or designed in this study were donated to the stock centers: *PEC1ox-3* (N2112900), *PEC1ox-6* (N2112901), *PEC1ox-11* (N2112902), *PEC1ox-6* sAEQ (N2112905), *PEC1ox-11* sAEQ (N2112906), *pec1pec2-1* pMAQ2 (N2112907), *pec1pec2-2* pMAQ2 (N2112908), *fou2pec1pec2-1* (N2112898), *fou2pec1pec2-2* (N2112899), *fou2* sAEQ (N2112903), and *fou2pec1pec2-1* sAEQ (N2112904). Further detailed description of the materials and methods used in this study are provided in the *SI Appendix*. In brief, *Arabidopsis* plant material and growth conditions, the generation of transgenic lines, chloroplast isolation, electrophysiological measurements on isolated chloroplasts, measurement of Ca^2+^ transients, various stress treatments, protein extraction, and immunoblotting are described in detail. The *SI Appendix* further includes information on the reanalysis of publicly available DAP-Seq, ChIP-Seq, RNA-seq, and spatiotemporal single-cell RNA-Seq data, quantification of luciferase luminescence, RNA Sequencing, and data analysis, as well as fresh weight determination, quantification of photosynthetic parameters, and leaf chlorophyll determination. Detailed procedures for whole rosette and chloroplast ionomics, the *B. cinerea* infection assay and Trypan Blue staining, root length analysis, and phytohormone quantification are also included. The applications used for data processing and analysis are also provided in the *SI Appendix*. Information on the respective tests applied, *P* values, and numbers of replicates for each experiment are indicated in the figure captions.

## Supplementary Material

Appendix 01 (PDF)

Dataset S01 (XLSX)

Dataset S02 (XLSX)

Dataset S03 (XLSX)

Dataset S04 (XLSX)

Dataset S05 (XLSX)

Dataset S06 (XLSX)

Dataset S07 (XLSX)

Dataset S08 (XLSX)

Dataset S09 (XLSX)

Dataset S10 (XLSX)

## Data Availability

Study data are included in the article and/or supporting information. Raw sequencing data have been deposited with NCBI SRA under the bioproject accessions PRJNA1268713 (63) for Dataset S1 and PRJNA1464431 (64) for Dataset S2.

## References

[1] Z. He, S. Webster, S. Y. He, Growth–defense trade-offs in plants. Curr. Biol. **32**, R634–R639 (2022). 35728544 10.1016/j.cub.2022.04.070

[2] R. K. Monson, A. M. Trowbridge, R. L. Lindroth, M. T. Lerdau, Coordinated resource allocation to plant growth–defense tradeoffs. New Phytol. **233**, 1051–1066 (2022). 34614214 10.1111/nph.17773

[3] J. I. Schroeder , Using membrane transporters to improve crops for sustainable food production. Nature **497**, 60–66 (2013). 23636397 10.1038/nature11909 PMC3954111

[4] T. T. Ho, H. N. Murthy, S. Y. Park, Methyl Jasmonate induced oxidative stress and accumulation of secondary metabolites in plant cell and organ cultures. Int. J. Mol. Sci. **21**, 716 (2020). 31979071 10.3390/ijms21030716 PMC7037436

[5] P. Köster, T. A. DeFalco, C. Zipfel, Ca^2+^ signals in plant immunity. EMBO J. **41**, e110741 (2022). 35560235 10.15252/embj.2022110741 PMC9194748

[6] M. Toyota , Glutamate triggers long-distance, calcium-based plant defense signaling. Science **361**, 1112–1115 (2018). 30213912 10.1126/science.aat7744

[7] E. E. Farmer, Y.-Q. Gao, G. Lenzoni, J.-L. Wolfender, Q. Wu, Wound- and mechanostimulated electrical signals control hormone responses. New Phytol. **227**, 1037–1050 (2020). 32392391 10.1111/nph.16646

[8] A. H. Howell , Pavement cells distinguish touch from letting go. Nat. Plants **9**, 877–882 (2023). 37188852 10.1038/s41477-023-01418-9

[9] M. K. Meena , The Ca2+ channel CNGC19 regulates *Arabidopsis* defense against *Spodoptera* herbivory. Plant Cell **31**, 1539–1562 (2019). 31076540 10.1105/tpc.19.00057 PMC6635850

[10] T. R. Vincent , Interplay of plasma membrane and vacuolar ion channels, together with BAK1, elicits rapid cytosolic calcium elevations in *Arabidopsis* during aphid feeding. Plant Cell **29**, 1460–1479 (2017). 28559475 10.1105/tpc.17.00136 PMC5502460

[11] C. Yan , Injury activates Ca(2+)/calmodulin-dependent phosphorylation of JAV1-JAZ8-WRKY51 complex for jasmonate biosynthesis. Mol. Cell **70**, 136–149.e37 (2018). 29625034 10.1016/j.molcel.2018.03.013

[12] G. A. Howe, I. T. Major, A. J. Koo, Modularity in jasmonate signaling for multistress resilience. Annu. Rev. Plant Biol. **69**, 387–415 (2018). 29539269 10.1146/annurev-arplant-042817-040047

[13] P. Armengaud, R. Breitling, A. Amtmann, The potassium-dependent transcriptome of Arabidopsis reveals a prominent role of jasmonic acid in nutrient signaling. Plant Physiol. **136**, 2556–2576 (2004). 15347784 10.1104/pp.104.046482 PMC523322

[14] E. Bell, R. A. Creelman, J. E. Mullet, A chloroplast lipoxygenase is required for wound-induced jasmonic acid accumulation in *Arabidopsis*. Proc. Natl. Acad. Sci. U.S.A. **92**, 8675–8679 (1995). 7567995 10.1073/pnas.92.19.8675 PMC41029

[15] C. Wasternack, B. Hause, Jasmonates: Biosynthesis, perception, signal transduction and action in plant stress response, growth and development. An update to the 2007 review in Annals of Botany. Ann. Bot. **111**, 1021–1058 (2013). 23558912 10.1093/aob/mct067 PMC3662512

[16] A. Mine , An incoherent feed-forward loop mediates robustness and tunability in a plant immune network. EMBO Rep. **18**, 464–476 (2017). 28069610 10.15252/embr.201643051 PMC5331241

[17] D. Maynard , Biochemical characterization of 13-Lipoxygenases of Arabidopsis thaliana. Int. J. Mol. Sci. **22**, 10237 (2021). 34638573 10.3390/ijms221910237 PMC8508710

[18] S. Mochizuki, K. Matsui, Green leaf volatile-burst in *Arabidopsis* is governed by galactolipid oxygenation by a lipoxygenase that is under control of calcium ion. Biochem. Biophys. Res. Commun. **505**, 939–944 (2018). 30309649 10.1016/j.bbrc.2018.10.042

[19] H. Morin , Wound-response jasmonate dynamics in the primary vasculature. New Phytol. **240**, 1484–1496 (2023). 37598308 10.1111/nph.19207

[20] K. Pappan, L. Zheng, R. Krishnamoorthi, X. Wang, Evidence for and characterization of Ca2+ binding to the catalytic region of *Arabidopsis thaliana* phospholipase Dβ*. J. Biol. Chem. **279**, 47833–47839 (2004). 15356005 10.1074/jbc.M402789200

[21] F. Resentini, C. Ruberti, M. Grenzi, M. C. Bonza, A. Costa, The signatures of organellar calcium. Plant Physiol. **187**, 1985–2004 (2021). 33905517 10.1093/plphys/kiab189 PMC8644629

[22] S. Stael , Plant organellar calcium signalling: An emerging field. J. Exp. Bot. **63**, 1525–1542 (2012). 22200666 10.1093/jxb/err394 PMC3966264

[23] G. Kreimer, M. Melkonian, J. A. Holtum, E. Latzko, Stromal free calcium concentration and light-mediated activation of chloroplast fructose-1, 6-bisphosphatase. Plant Physiol. **86**, 423–428 (1988). 16665924 10.1104/pp.86.2.423 PMC1054500

[24] G. Lenzoni, M. R. Knight, Increases in absolute temperature stimulate free calcium concentration elevations in the chloroplast. Plant Cell Physiol. **60**, 538–548 (2019). 30517735 10.1093/pcp/pcy227

[25] H. Nomura , Chloroplast-mediated activation of plant immune signalling in Arabidopsis. Nat. Commun. **3**, 926 (2012). 22735454 10.1038/ncomms1926

[26] A. K. Hochmal, S. Schulze, K. Trompelt, M. Hippler, Calcium-dependent regulation of photosynthesis. Biochim. Biophys. Acta **1847**, 993–1003 (2015). 25687895 10.1016/j.bbabio.2015.02.010

[27] H.-H. Kunz, U. Armbruster, S. Mühlbauer, J. de Vries, G. A. Davis, Chloroplast ion homeostasis–What do we know and where should we go? New Phytol. **243**, 543–559 (2024). 38515227 10.1111/nph.19661

[28] E. Teardo , A chloroplast-localized mitochondrial calcium uniporter transduces osmotic stress in *Arabidopsis*. Nat. Plants **5**, 581–588 (2019). 31182842 10.1038/s41477-019-0434-8

[29] X. Zhang , Perturbation of mitochondrial Ca2+ homeostasis activates cross-compartmental proteostatic response in *Arabidopsis*. Stress Biol. **6**, 40 (2026). 42207405 10.1007/s44154-026-00314-4 PMC13219543

[30] J. Frank , Chloroplast-localized BICAT proteins shape stromal calcium signals and are required for efficient photosynthesis. New Phytol. **221**, 866–880 (2019). 30169890 10.1111/nph.15407

[31] I. I. Pottosin, J. Muñiz, S. Shabala, Fast-activating channel controls cation fluxes across the native chloroplast envelope. J. Membr. Biol. **204**, 145–156 (2005). 16245037 10.1007/s00232-005-0758-3

[32] I. Pottosin, O. Dobrovinskaya, Ion channels in native chloroplast membranes: Challenges and potential for direct patch-clamp studies. Front. Physiol. **6**, 396 (2015). 26733887 10.3389/fphys.2015.00396 PMC4686732

[33] C. Völkner , Two plastid POLLUX ion channel-like proteins are required for stress-triggered stromal Ca2+ release. Plant Physiol. **187**, 2110–2125 (2021). 34618095 10.1093/plphys/kiab424 PMC8644588

[34] R. M. Lilley, M. P. Fitzgerald, K. G. Rienits, D. A. Walker, Criteria of intactness and the photosynthetic activity of spinach chloroplast preparations. New Phytol. **75**, 1–10 (1975).

[35] M. R. Knight, A. K. Campbell, S. M. Smith, A. J. Trewavas, Transgenic plant aequorin reports the effects of touch and cold-shock and elicitors on cytoplasmic calcium. Nature **352**, 524–526 (1991). 1865907 10.1038/352524a0

[36] I. López-Vidriero , DNA features beyond the transcription factor binding site specify target recognition by plant MYC2-related bHLH proteins. Plant Commun. **2**, 100232 (2021). 34778747 10.1016/j.xplc.2021.100232 PMC8577090

[37] R. C. O’Malley , Cistrome and epicistrome features shape the regulatory DNA landscape. Cell **165**, 1280–1292 (2016). 27203113 10.1016/j.cell.2016.04.038 PMC4907330

[38] M. Boter, O. Ruíz-Rivero, A. Abdeen, S. Prat, Conserved MYC transcription factors play a key role in jasmonate signaling both in tomato and Arabidopsis. Genes Dev. **18**, 1577–1591 (2004). 15231736 10.1101/gad.297704 PMC443520

[39] P. Fernández-Calvo , The *Arabidopsis* bHLH transcription factors MYC3 and MYC4 are targets of JAZ repressors and act additively with MYC2 in the activation of jasmonate responses. Plant Cell **23**, 701–715 (2011). 21335373 10.1105/tpc.110.080788 PMC3077776

[40] A. Van Moerkercke , A MYC2/MYC3/MYC4-dependent transcription factor network regulates water spray-responsive gene expression and jasmonate levels. Proc. Natl. Acad. Sci. U.S.A. **116**, 23345–23356 (2019). 31662474 10.1073/pnas.1911758116 PMC6859355

[41] M. Zander , Integrated multi-omics framework of the plant response to jasmonic acid. Nat. Plants. **6**, 290–302 (2020). 32170290 10.1038/s41477-020-0605-7 PMC7094030

[42] D. M. Brooks, C. L. Bender, B. N. Kunkel, The *Pseudomonas syringae* phytotoxin coronatine promotes virulence by overcoming salicylic acid-dependent defences in *Arabidopsis thaliana*. Mol. Plant Pathol. **6**, 629–639 (2005). 20565685 10.1111/j.1364-3703.2005.00311.x

[43] R. Hickman , Architecture and dynamics of the jasmonic acid gene regulatory network. Plant Cell **29**, 2086–2105 (2017). 28827376 10.1105/tpc.16.00958 PMC5635973

[44] S. Zenker, K. Schiller, A. Bräutigam, Time- and space-resolved transcriptional regulation in Arabidopsis thaliana. Plant Biol. (Stuttg) **28**, 1005–1015 (2026). 41827035 10.1111/plb.70196 PMC13175949

[45] G. Glauser , Velocity estimates for signal propagation leading to systemic jasmonic acid accumulation in wounded *Arabidopsis*. J. Biol. Chem. **284**, 34506–34513 (2009). 19846562 10.1074/jbc.M109.061432 PMC2787311

[46] P. E. Staswick, W. Su, S. H. Howell, Methyl jasmonate inhibition of root growth and induction of a leaf protein are decreased in an *Arabidopsis thaliana* mutant. Proc. Natl. Acad. Sci. U.S.A. **89**, 6837–6840 (1992). 11607311 10.1073/pnas.89.15.6837 PMC49599

[47] C. Ellis, J. G. Turner, A conditionally fertile coi1 allele indicates cross-talk between plant hormone signalling pathways in Arabidopsis thaliana seeds and young seedlings. Planta **215**, 549–556 (2002). 12172836 10.1007/s00425-002-0787-4

[48] G. Bonaventure , A gain-of-function allele of TPC1 activates oxylipin biogenesis after leaf wounding in Arabidopsis. Plant J. **49**, 889–898 (2007). 17253984 10.1111/j.1365-313X.2006.03002.x

[49] S. Schwartz , Catch & Release–Rapid cost-effective protein purification from plants using a DIY GFP-Trap-protease approach. bioXriv [Preprint] (2025). 10.1101/2025.05.15.654187. PMC12611452 41222448

[50] S. Nagaya, K. Kawamura, A. Shinmyo, K. Kato, The HSP terminator of *Arabidopsis thaliana* increases gene expression in plant cells. Plant Cell Physiol. **51**, 328–332 (2010). 20040586 10.1093/pcp/pcp188

[51] Y. Zhang, J. G. Turner, Wound-induced endogenous jasmonates stunt plant growth by inhibiting mitosis. PLoS One **3**, e3699 (2008). 19002244 10.1371/journal.pone.0003699 PMC2577035

[52] L. J. Holzner , The chloroplast ionome shines light on the dynamics of organellar iron homeostasis. Plant Cell **38**, koag017 (2026). 41610165 10.1093/plcell/koag017

[53] G. R. Littlejohn, S. Breen, N. Smirnoff, M. Grant, Chloroplast immunity illuminated. New Phytol. **229**, 3088–3107 (2021). 33206379 10.1111/nph.17076

[54] A. Bittner , Organelles and phytohormones: A network of interactions in plant stress responses. J. Exp. Bot. **73**, 7165–7181 (2022). 36169618 10.1093/jxb/erac384 PMC9675595

[55] E. S. Haswell, E. M. Meyerowitz, MscS-like proteins control plastid size and shape in *Arabidopsis thaliana*. Curr. Biol. **16**, 1–11 (2006). 16401419 10.1016/j.cub.2005.11.044

[56] C. Völkner , Evidence for partial functional overlap of KEA and MSL transport proteins in the chloroplast inner envelope of *Arabidopsis thaliana*. FEBS Lett. **598**, 1877–1887 (2024). 38658177 10.1002/1873-3468.14887

[57] A. Choudhary, M. Ammari, Hyuk S. Yoon, M. Zander, High-throughput capture of transcription factor-driven epigenome dynamics using PHILO ChIP-seq. Nucleic Acids Res. **52**, e105 (2024). 39588772 10.1093/nar/gkae1123 PMC11662648

[58] S. W. Wilkinson , Long-lasting memory of jasmonic acid-dependent immunity requires DNA demethylation and ARGONAUTE1. Nat. Plants **9**, 81–95 (2023). 36604579 10.1038/s41477-022-01313-9

[59] S. A. R. Mousavi, A. Chauvin, F. Pascaud, S. Kellenberger, E. E. Farmer, Glutamate receptor-like genes mediate leaf-to-leaf wound signalling. Nature **500**, 422–426 (2013). 23969459 10.1038/nature12478

[60] M. Walther, R. Wiesner, H. Kuhn, Investigations into Calcium-dependent Membrane Association of 15-Lipoxygenase-1: MECHANISTIC ROLES OF SURFACE-EXPOSED HYDROPHOBIC AMINO ACIDS AND CALCIUM*. J. Biol. Chem. **279**, 3717–3725 (2004). 14594811 10.1074/jbc.M309564200

[61] S. A. Tatulian, J. Steczko, W. Minor, Uncovering a calcium-regulated membrane-binding mechanism for soybean lipoxygenase-1. Biochemistry **37**, 15481–15490 (1998). 9799511 10.1021/bi981062t

[62] C. Ellis, I. Karafyllidis, C. Wasternack, J. G. Turner, The Arabidopsis mutant cev1 links cell wall signaling to jasmonate and ethylene responses. Plant Cell **14**, 1557–1566 (2002). 12119374 10.1105/tpc.002022 PMC150706

[63] S. Mühlbauer , RNAseq of Arabidopsis thaliana Col-0 and pec1pec2 mutant plants subjected to wounding. NCBI BioProject. https://www.ncbi.nlm.nih.gov/bioproject/PRJNA1268713. Deposited 28 May 2025.

[64] S. Mühlbauer , mRNAseq of wounded PEC1 over-expression and WT Arabidopsis thaliana plants. NCBI BioProject. https://www.ncbi.nlm.nih.gov/bioproject/PRJNA1464431. Deposited 11 May 2026.

