# Supplementary material for: PLASTID ENVELOPE ION CHANNELS (PEC1/2) link Ca2+ and jasmonic acid signaling in plant cells
Source: Proc Natl Acad Sci U S A. 2026 Aug 28;123(35):e2525536123. doi: 10.1073/pnas.2525536123 (PMC13535249; doi:10.1073/pnas.2525536123)
Supplement: Supplementary file 1 [file pnas.2525536123.sapp.pdf]

PNAS

Supporting Information for
PLASTID ENVELOPE ION CHANNELS (PEC1/2) link Ca^{2+} and jasmonic acid signaling in plant cells

Susanne Mühlbauer[a], Dawid Jaślan[b], Inês F. Duarte Nunes[a], Benjamin Brandt[a], Lena Wutz[a], Khansa Mekkaoui[c], Sanja Zenker[d,e], Jakob Rehberger[a], Marlena Rädler[a], Lorenz Holzner[a], Constance Tisserant[f], Carsten Völkner[a], Andrea Bräutigam[d,e], Silke Robatzek[f], Bettina Hause[c], Christian Grimm[b,g,h], and Hans-Henning Kunz[a]*

[a] Plant Biochemistry and Physiology, Faculty of Biology, Ludwig-Maximilians-University Munich, Planegg-Martinsried 82152, Germany
[b] Walther Straub Institute of Pharmacology and Toxicology, Faculty of Medicine, Ludwig-Maximilians-University Munich, Munich 80336, Germany
[c] Department of Cell and Metabolic Biology, Leibniz Institute of Plant Biochemistry, Halle/Saale 06120, Germany
[d] Computational Biology, Faculty of Biology, Bielefeld University, Bielefeld 33615, Germany
[e] Department of Computational Biology, Center of Biotechnology, Bielefeld University, Bielefeld 33615, Germany
[f] Genetics, Faculty of Biology, Ludwig-Maximilians-University Munich, Planegg-Martinsried 82152, Germany
[g] Department of Pharmacology, Faculty of Medicine, University of Oxford, Oxford OX1 3QT, United Kingdom
[h] Immunology, Infection and Pandemic Research, Fraunhofer Institute for Translational Medicine and Pharmacology, Munich/Frankfurt 80799, Germany

*correspondence: Hans-Henning Kunz
Email: kunz@lmu.de

This PDF file includes:

Supporting text – SI Materials and Methods
Figures S1 to S13
Legends for Datasets S1 to S10
SI references

Other supporting materials for this manuscript include the following:

Datasets S1 to S10

Supporting Information Text

SI Materials and Methods

Plant material and growth conditions

A. thaliana ecotype Col-0 or gl-1 (*coi1-16*) were grown in long-day conditions if not stated otherwise (16 h illumination / 8 h dark, 110 µmol photons m^{-2} s^{-1}, 50 % humidity) on soil (Substrate A210, Stender, Schermbeck, Germany), ½ Murashige & Skoog (MS) plates (1) containing 0.8 % (w/v) Phytoagar, or liquid ½ MS supplemented with 1 % (w/v) Sucrose as indicated for each experiment. For sterile conditions, seeds were surface-sterilized with 70 % (v/v) ethanol, followed by 99.8 % (v/v) ethanol before sowing. Seeds were stratified at 4 °C for at least 48 h. Specific growth substrates and plant ages are indicates in the respective experimental sections.
To obtain higher order mutants, the respective single and/or higher order mutant alleles were crossed. All genotypes were confirmed by PCR using primer combinations listed in Dataset S10 (Fig. S13).

Generation of transgenic lines

The native *AtPEC1* promoter and the *AtPEC1* coding sequence was amplified by PCR with gene-specific primers (Dataset S10). PCR products were cloned via Gibson assembly into the pGreen 2.1 system containing a Fluorescence-Accumulating Seed Technology (FAST) red selection cassette and *HSP18.2* terminator (2). *A. thaliana pec1pec2* loss-of-function mutants were transformed via floral dip (3) using the GV3101 agrobacteria strain supplemented with pSOUP helper plasmid (4). Transgenic plants were selected in T_1 by screening for RFP fluorescence in the seed coat. Additionally, immunoblotting was carried out on these individuals to identify PEC1 overaccumulators.

Chloroplast isolation

Chloroplasts were isolated as described by Aronsson and Jarvis (5) with minor modifications. In brief, 21-day-old Arabidopsis grown on soil were dark-adapted for 24 h, harvested, and homogenized in pre-cooled isolation buffer (300 mM sorbitol, 5 mM $MgCl_2$, 5 mM EDTA, 20 mM HEPES/NaOH pH 8.0, 50 mM ascorbic acid, 0.1 % (W/v) BSA). Homogenization was performed using a Politron homogenizer (Bachhofer, Reutlingen, Germany). The homogenate was filtered through gauze and centrifuged at 1500*g* for 3 min at 4 °C. The pellet was resuspended in isolation buffer and layered onto an 82 % (v/v) / 30 % (v/v) two-step Percoll gradient (82% (v/v)/30% (v/v) Percoll (GE Healthcare, Chicago, USA), 300 mM sorbitol, 5 mM $MgCl_2$, 5 mM EDTA, 20 mM HEPES/NaOH pH 8.0). Gradients were centrifuged at 2000*g* for 10 min at 4°C. Intact chloroplasts were collected from the interface, washed twice in chloroplast washing buffer (300 mM sorbitol, 5 mM $MgCl_2$, 5 mM EDTA, 20 mM HEPES/NaOH pH 8.0), and pelleted at 1000*g* for 5 min at 4°C. Chlorophyll concentration was determined in 80% (v/v) acetone by measuring absorbance at 645 nm, 663 nm, and 750 nm (6). Chlorophyll concentration was calculated according to the following equation:

µg chlorophyll / µL sample = 8.02 x (A645 nm – A750 nm) + 20.2 x (A663 nm – A750 nm).

Electrophysiological measurements on isolated chloroplasts

For manual inside (stromal side)-out chloroplast patch-clamp recordings (7), freshly isolated chloroplasts from adult *Pisum sativum* and 3-week-old Arabidopsis plants grown on soil were used. For Arabidopsis, experiments were carried out double-blind. Both the cytoplasmic and pipette solutions contained 250 mM KCl, 2 mM $CaCl_2$, 2 mM $MgCl_2$, and 10 mM HEPES (pH adjusted to 7.3 with KOH, osmolality to 800 mOsm with sorbitol) for general recordings. To test for cation or anion specificity of PECs, 250 mM KCl was substituted for 250 mM potassium gluconate in both cytoplasmic and pipette solutions. Recording pipettes were pulled from borosilicate glass and polished to resistances in the range of 8-11 MΩ. Gd^{3+} application was carried out by replacing the cytoplasmic solution with a freshly diluted $GdCl_3$-containing solution at 100 µM. Data acquisition and digitization (40 kHz) were performed with an EPC-10 amplifier and PatchMaster software (both HEKA, Lambrecht, Germany), with a low-pass filter frequency of 2.9 kHz. Recurring 5 s voltage ramps (from -150 mV to +150 mV) ended with 100 ms - 60 mV step was applied; quantified currents

were extracted from individual ramps at -150 mV and +150 mV. Data were statistically analyzed using one-way ANOVA or paired t-tests, with offline analyses performed in GraphPad Prism 10.2.3.

Measurement of Ca^{2+} transients
Cytosolic and stromal Ca^{2+} transients were measured in stable Arabidopsis aequorin reporter lines. For cytosolic Ca^{2+} measurements, homozygous *pec1pec2* loss-of-function lines carrying cytosolic aequorin were generated by crossing pMAQ2 (8) with both *pec1pec2-1* and *pec1pec2-2* mutants and selecting homozygous individuals by genotyping in F_3. For stromal Ca^{2+} measurements, a homozygous WT and the *pec1pec2-1* reporter line expressing stromal-targeted YFP-aequorin (NTRC-YA) (9, 10) were crossed into *fou2* mutants. Additionally, *pec1pec2-2* expressing NTRC-YA was crossed with the three *PEC1* overexpression lines (*PEC1ox-3*, *PEC1ox-6*, *PEC1ox-11*). Homozygosity was confirmed by PCR and phenotyping in the F_3 generation. Aequorin assays were performed as described previously (11), with minor modifications to the CTZ buffer. In brief, 5-day-old seedlings grown sterile on ½ MS plates were transferred into white 96-well plates containing 50 µL of CTZ buffer (1.4 mM $CaCl_2$, 20 mM KCl, 5 mM MES-KOH pH 5.7) supplemented with 10 µM Coelenterazine (Cayman Chemical, Ann Arbor, USA). For MeJA treatment, 5-day-old seedlings were transferred to 96-well-plates containing 50 µL of CTZ buffer supplemented with 200 µM of MeJA (Sigma-Aldrich, St. Louis, USA). Seedlings were incubated overnight in the dark at 22°C to allow reconstitution of aequorin. Luminescence was recorded using a TECAN Spark plate reader (Tecan, Männedorf, Switzerland) with an integration time of 500 ms. For each well, total aequorin luminescence capacity was determined by discharging with 10% (v/v) ethanol and 1 M $CaCl_2$. Calculations were performed as described by Knight, Trewavas and Knight (12).

Stress treatments
Arabidopsis seedlings grown on ½ MS plates for 14 days were treated by either application of MeJA or COR or by mechanical wounding (two squeezes per leaf with a fine tweezer) for a maximum of 96 h. For MeJA and COR treatments, seedlings were transferred to ½ MS plates supplemented with 20 µM MeJA or 1 µM COR (Sigma-Aldrich, St. Louis, USA), respectively. As a control, seedlings were transferred to ½ MS plates containing neither COR, nor MeJA. For analysis of sustained response (96 h) after wounding, 3-week-old plants grown on soil were wounded twice on three adult leaves perpendicularly to the midrib. Plants were harvested at the indicated time points for downstream analysis.

Protein extraction and immunoblotting
Total protein extraction and immunoblotting were carried out as described previously (9, 13). Plant material was ground in liquid nitrogen, normalized by fresh weight, and subsequently homogenized and solubilized for 30 min at 4°C in solubilization buffer (10% (v/v) glycerol, 150 mM NaCl, 2 mM EDTA, 200 mM DTT, 50 mM Tris-HCl pH 7.5, 1% (w/v) SDS, 1X plant protease inhibitor mix (PI) (Sigma-Aldrich, St. Louis, USA)). Subsequently, samples were mixed with SDS loading dye (62.5 mM Tris-HCl pH 6.8, 2% (w/v) SDS, 10% (v/v) glycerol, 0.003% (w/v) bromophenol blue) and boiled for 10 min at 80°C. Proteins were separated by SDS-PAGE (10% acrylamide) and transferred to PVDF membranes. Blots were incubated overnight at 4°C with the respective primary antibodies (PEC1 (9), LOX-c: AS07 258, ACTIN: AS13 2640). For detection, blots were incubated with horse radish peroxidase (HRP)-coupled secondary antibodies and developed using enhanced chemiluminescence (ECL) with an ImageQuant LAS 4000 system (GE Healthcare, Chicago, USA). Fiji (14) was used to quantify band intensities.

Re-analysis of publicly available DAP-Seq data
Promoter regions of *PEC1/2* were defined as 2 kb upstream of the transcription start site. Processed DAP-Seq binding data was retrieved from GSE155321 (15) and GSE60143 (16) and visualized using ggplot. Signal values were normalized within each TF experiment. Gene ontology enrichment was analyzed with g:Profiler with g:SCS ($p < 0.05$) (17). Excluding GO terms associated with DNA binding, resulting reduced GO networks were condensed using Revigo (18) and further processed with Cytoscape (19).

Re-analysis of publicly available ChIP-Seq data
ChIP-Seq data focusing on MYC2 targets was retrieved from Van Moerkercke et al. (20), GSE132316. FASTQ files were downloaded and processed on usegalaxy.eu (21). Reads (single-end, Illumina platform) were aligned to the *A. thaliana* TAIR10 genome (22) using BWA-MEM (23) default parameters, the output sorted by chromosomal coordinates in BAM format. BAM files from untreated and treated samples were compared to control samples using bamCompare (24) with a bin size if 1 bp, read count normalization, and ratio mode (without exact scaling factor). Analyses were restricted to genomic loci of *PEC1* (Chr5:683630-691192) and *PEC2 (Chr5:17569000-17580000)*. Bedgraph files generated by bamCompare were visualized in GraphPad Prism.

Re-analysis of publicly available RNA-seq data
Reads were downloaded from the NCBI SRA Bioproject PRJNA224133 (25) and mapped with kallisto (26) to the *A. thaliana* TAIR10 transcriptome (22).

Re-analysis of publicly available spatio-temporal single-cell RNA-Seq data
To assess spatiotemporal expression patterning of key transcripts, transcriptomic data of 3-week-old *Arabidopsis thaliana* above ground material data was visualized according to Zenker, Schiller and Bräutigam (27). Processed data of 3-week-old *A. thaliana* sampled every 2 h over 24 h was retrieved from Zenker, Schiller and Bräutigam (27) and paired with single cell RNA-seq data of 3-week-old *A. thaliana* rosettes from Lee*, et al.* (28).

Quantification of luciferase luminescence
For luciferase imaging, 3-week-old Arabidopsis grown on soil were sprayed with 50 µM D-luciferin (BioVision, Milpitas, USA) in 0.01% (v/v) Tween-20 16 h prior to imaging. During the experiment, plants were either wounded (three squeezes per leaf parallel to the midrib using a tweezer), or sprayed with 200 µM MeJA or 10 µM COR. Luminescence was recorded using a Photek camera system for 3.5 h post-treatment, and accumulated photon counts were visualized using Image32 (both Photek Ltd., St. Leonards-on-Seq, UK).

RNA Sequencing and data analysis
For RNA-Seq dataset 1, RNA-Seq was performed on 3-week-old soil-grown Arabidopsis plants harvested 90 min after wounding (three squeezes on three mature leaves per plant parallel to the midrib) and immediately flash frozen in liquid nitrogen. Total RNA was extracted using the RNeasy Plant Mini Kit (Qiagen, Hilden, Germany) with on-column DNase digestion according to the manufacturer's instructions. For RNA-Seq dataset 2, 3-week-old WT, *PEC1ox-6* and *PEC1ox-11* were wounded three times parallel to the midrip on leaves 5, 6, and 7. Plants were sampled 48 h after the initial wounding (48 h Primed). In addition, a subset of 48 h primed plants was wounded again on leaves 7, 8, and 9 and sampled 90 min later to assess the acute wounding response (48 h Wound). To investigate prolonged priming, another subset of primed plants received a second wounding on leaves 7, 8, and 9 48 h after the first wounding, and was harvested 48 h later, corresponding to 96 h after the initial wounding (96 h Primed). Finally, 96 h primed plants were wounded a third time on leaves 8, 9, and 10 and sampled 90 min later (96 h Wound). Unwounded control plants were harvested for all genotypes and time points in parallel. RNA was extracted using the innuPREP Plant RNA - PPMini Kit (IST Innuscreen, Berlin, Germany) and the KingFisher Apex (Thermo Fisher Scientific, Waltham, USA). After passing RNA quality controls, RNA-Seq libraries were prepared and sequenced (150 bp paired-end reads) by BMKGENE (Münster, Germany).
Analysis of both RNA-Seq datasets was carried out as previously described in Krämer*, et al.* (29) with minor modifications. Unless stated otherwise, the analysis was carried out on the Galaxy platform (21) using standard settings. Reads were trimmed using TrimGalore! (Version 0.6.7; https://https://github.com/FelixKrueger/TrimGalore.com/fenderglass/Flye), quality-checked using FastQC (Version 0.11.9; https://www.bioinformatics.babraham.ac.uk/projects/fastqc/) and subsequently mapped to the TAIR10 reference genome (22) using STAR (Version 2.7.8a; (30)). During the mapping for dataset 2 we realized that for 8 samples an unusually small percentage of reads mapped to the Arabidopsis genome. Further inspection revealed that the residual reads mapped to the ArLV1 virus, which does not elicit any phenotypes or changes in the transcriptome (31, 32). These libraries were further sequenced that > 10 M reads were mapped to the Arabidopsis

genome.The high quality of the mapping was confirmed using the RseQC software package (Version 5.0.1;(33)). Reads mapped to genes were counted with featureCounts (Version 2.0.1;(34)) using the TAIR10 gene annotation (Arabidopsis_thaliana.TAIR10.52.gtf downloaded from ensembl.org).

Read count normalization, statistical analysis and assessment of differentially expressed genes was performed with the limma-voom package which includes EdgeR (35-37). Low read counts were filtered with at least 1 counts per million in at least 1 sample, read counts were normalized with TMM, and differentially expressed genes were assessed with a one factor analysis. Statistical cutoffs are indicated in the figure legends.

For dataset 1, the wounding only read count table of the feature counts tool, k-means and hierarchical clustering was carried out using the iDEP platform (Version 2.01) (38). Here, besides differences stated in the following standard setting were used: Low read counts were removed retaining genes with at least 0.5 cpm in 2 libraries. K-means clustering of the top 2000 diverse genes by standard deviation of wounding only samples was performed setting the numbers of clusters to 6, centering and normalizing genes. Average Pearson hierarchical clustering of the top 2000 diverse genes by standard deviation was performed with centered and normalized genes.

Euclidean hierarchical row clustering of z-scored normalized read count of dataset 2 was carried out in the Perseus software (39). Gene ontology enrichment was analyzed with g:Profiler (17) with Benjamini-Hochberg FDR or gSCS corrected *p* values (as indicated in the figure legends. PCA and bubbles plots for the GO-term enrichment were plotted using the ggplot2 R package (40). Venn diagrams were created using the JVenn or DiVenn softwares (41, 42).

Fresh weight determination

Whole rosettes of 3-week-old plants grown on soil were harvested and weighed using the Cubis® individual balance (Sartorius, Göttingen, Germany).

Quantification of photosynthetic parameters

Photosynthetic parameters were assessed via pulse-amplitude modulated (PAM) chlorophyll fluorescence measurements with a Walz IMAGING-PAM M-Series MAXI version (Walz, Effeltrich, Germany) as described previously (43). In brief, 3 week-old plants grown on soil were dark-adapted for 30 min prior to measurement. Standard induction curves were recorded at 110 µmol photons m^{-2} s^{-1} for 300 s in 20 s steps. The data was processed using the ImagingWin software (Walz, Effeltrich, Germany).

Leaf chlorophyll determination

Leaf chlorophyll content was determined in 80% (v/v) acetone according to Lichtenthaler (44) and Porra, Thompson and Kriedemann (45). In brief, chlorophyll of 20 mg of ground leaf tissue was extracted in 80% (v/v) acetone overnight at 4°C. Debris was removed by centrifugation at 10,000*g* for 5 min at 4°C. Absorbance of the supernatant was measured at 663 nm and 646 nm using a spectrophotometer. Chlorophyll a and Chlorophyll b content per g fresh weight was calculated using the following equations:

µg Chl a / gFW = (13.71 x A663 – 2.85 x A646) / mass FW

µg Chl b / gFW = (22.39 x A646 – 5.42 x A663) / mass FW

Ionomics

Elemental analysis was performed by total reflection X-ray fluorescence (TXRF) on a S4 T-STAR spectrometer (Bruker, Berlin, Germany) as described previously for leaf tissue (46) and for chloroplasts (47). In brief, chloroplasts were isolated and mixed 1:1 with element standards to a final concentration of 50 ppm Scandium (WL) and 1 ppm Gallium (Mo-K), 0.1% (w/v) polyvinyl alcohol (PVA). Leaf tissue was dried, ground in a zirconium mortar, and digested in 69% (v/v) HNO_3 (Sigma-Aldrich, St. Louis, USA) at 95°C for 90 min and mixed 1:10 with element standards to a final concentration of 50 ppm Scandium and 1 ppm Gallium. Samples were spotted on siliconized quartz glass carriers and measured at 50 kV (1000 s for chloroplasts, 600 s for leaf tissue). Background (chloroplast washing buffer or 60% HNO_3) was subtracted. Element concentrations were normalized to chlorophyll content (chloroplasts) or dry weight (leaf tissue).

Botrytis cinerea infection assay and Trypan Blue staining
B05.10 wild-type strain conidia were obtained by growing the fungus on solid rich medium (10 g l^{-1} malt extract, 4 g l^{-1} yeast extract, 4 g l^{-1} glucose, 15 g l^{-1} agar) for 2 weeks, at room temperature and under near-ultraviolet light exposure. Infection assays were performed as described previously (48) on detached leaves of 5-week old Arabidopsis plants grown on soil (12 h light / 12 h dark, 22°C, 60% humidity). Briefly, fully developed leaves (positions 8-12) were inoculated with 20 µL of conidia suspension (2 x 10^5 conidia/mL in 1% malt extract). Inoculated leaves were kept at room temperature in humidity boxes and under natural daylight. After 48 h, leaf discs (0.6 cm) were collected and stained with Trypan Blue (49). Microscopy was performed using a Leica stereoscope, and quantification of the lesion area was carried out with Fiji (14). After 72 h, all leaves were photographed and lesion sizes were measured with Fiji (14). Robust regression and Outlier removal (ROUT) (Q = 5%) was used to detect and remove statistical outliers. Significance was determined using Student's t-test. Additionally, representative leaves were selected for PAM analysis to visualize the lesions.

Root length analysis
Root growth was assessed as previously described by Staswick, Su and Howell (50), with minor modifications. For control conditions, Arabidopsis seedlings were grown vertically on ½ MS plates and root length was assessed after 5 days. To test for altered sensitivity to MeJA, 4-day old seedlings were transferred to ½ MS plates supplemented with 50 µM MeJA. Primary root length was measured 7 days after transfer. Images were analyzed using Fiji (14).

Phytohormone quantification
For hormone quantification in wounded plants, leaf number 8 from 5-week-old adult WT and the two *pec1pec2* loss-of-function mutants (10/14 h light/dark, soil-grown) was squeezed one time using a hemostat with serrated teeth 1 h prior to harvest. As controls, unwounded leaf 8 was collected. For priming experiments, seedlings grown in liquid ½ MS + 1% (w/v) sucrose were primed by gently squeezing grouped seedlings 3 to 4 times with a forceps on days 9 and 11 of development. Harsher 8x times squeezing was inflicted on day 14 or wounded samples 1 h before harvest while control seedlings were collected without wounding. Hormones were extracted and quantified as previously described by Mekkaoui*, et al.* (51). Quantification of *cis*-OPDA, JA, and JA-Ile was performed by UPLC-MS/MS (52). In brief, 50 mg frozen powdered plant material was extracted with 500 µL 100% LC-MS grade methanol containing 50 ng each of [$^2H_5$]OPDA, [$^2H_6$]JA, and [$^2H_2$]JA-Ile as internal standards. Samples were purified via solid-phase extraction on HR-XC columns (Chromabond, Macherey-Nagel, Düren, Germany). 10 µL of the eluate was analyzed by UPLC-MS/MS, and analyte concentrations were determined relative to internal standard peak heights.

Statistical analysis
Data was analyzed using GraphPad Prism (Dotmatics, Boston, USA). Significance was determined by one-way ANOVA followed by Tukey's multiple comparison test unless stated otherwise.
Figures were made using Affinity Designer 2 (Serif, Nottingham, UK).

Accession numbers
PEC1 (At5g02940), PEC2 (At5g43745), TPC1 (At4g03560), MYC2 (At1g32640), MYC3 (At5g46760), MYC4 (At4g17880), COI1 (At2g39940), LOX2 (At3g45140), JAR1 (At2g46370), *pec1-1pec2-1* (CS72541), *pec1-2pec2-2* (CS72542), *fou2* (53), *lox2-1* (54), *jar1-1* (CS8072), *myc234* (55), *coi1-16* (56), *fou2pec1pec2-1* (N2112898), *fou2pec1pec2-2* (N2112899), *PEC1ox-3* (N2112900), *PEC1ox-6* (N2112901), *PEC1ox-11* (N2112902), Col-0 WT sAEQ (CS72534), *pec1-1pec2-1* sAEQ (CS72535), *pec1-2pec2-2* sAEQ (CS72536), *fou2* sAEQ (N2112903), *fou2pec1pec2-1* sAEQ (N2112904), *PEC1ox-6* sAEQ (N2112905), *PEC1ox-11* sAEQ (N2112906), pMAQ2 (8), *pec1pec2-1* pMAQ2 (N2112907), *pec1pec2-2* pMAQ2 (N2112908).

AI use disclosure
ChatGPT (OpenAI) was used solely to assist with phrasing during the final revision of the manuscript and preparation of the response letter.

Figures

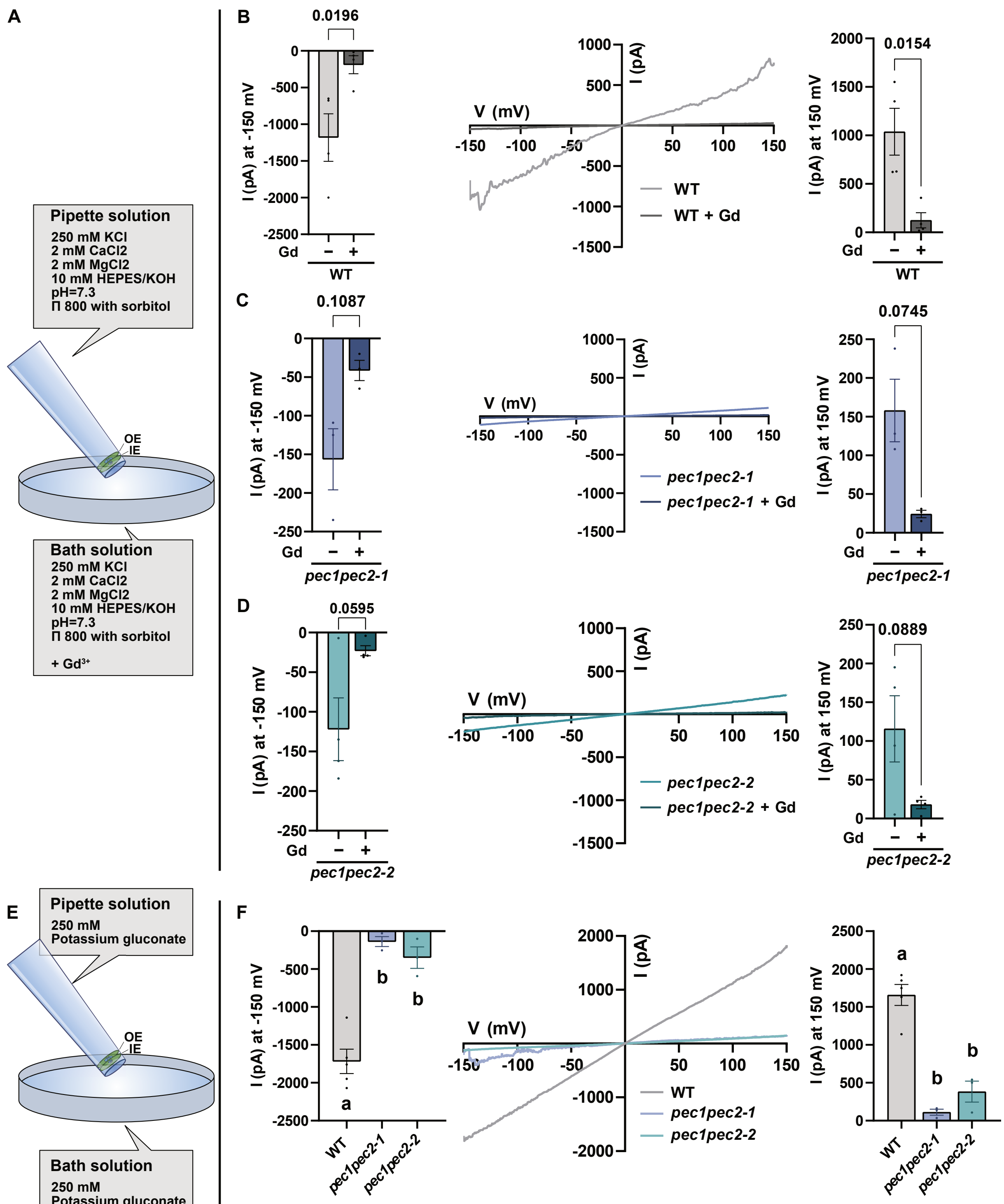

A
Pipette solution
250 mM KCl
2 mM CaCl2
2 mM MgCl2
10 mM HEPES/KOH
pH=7.3
Π 800 with sorbitol
OE
IE
Bath solution
250 mM KCl
2 mM CaCl2
2 mM MgCl2
10 mM HEPES/KOH
pH=7.3
Π 800 with sorbitol
+ Gd3+
B
0.0196
I (pA) at -150 mV
Gd
WT
V (mV)
I (pA)
WT
WT + Gd
0.0154
I (pA) at 150 mV
C
0.1087
pec1pec2-1
pec1pec2-1 + Gd
0.0745
D
0.0595
pec1pec2-2
pec1pec2-2 + Gd
0.0889
E
Pipette solution
250 mM
Potassium gluconate
Bath solution
250 mM
Potassium gluconate
F
a
b
b

Figure S1: Gd^{3+} sensitivity and cation selectivity support PECs as Arabidopsis mediators of FACC-like currents. (A) Experimental setup: Inside-out patch-clamp recordings of isolated chloroplasts were performed in symmetric 250 mM KCl. For unspecific blockage of ion channels, 100 µM $GdCl_3$ was added to the bath solution. (B) Gd^{3+} inhibits envelope currents in Arabidopsis WT chloroplasts. Representative I/V curves are shown, and quantified current amplitudes before and after application of 100 µM $GdCl_3$ at -150 mV and +150 mV are plotted (n = 4). Bars represent mean ± SEM; significance was determined via paired two-tailed t-test, **p* = 0.0196 (-150 mV), **p* = 0.0154 (+150 mV). (C, D) Application of 100 µM $GdCl_3$ to *pec1pec2* chloroplasts does not significantly alter currents. Representative I/V curves are shown for *pec1pec2-1* (C) and *pec1pec2-2* (D), and quantified current amplitudes at -150 mV and +150 mV are plotted, respectively (n = 3). Bars represent mean ± SEM; significance was determined via paired two-tailed t-test, *p* = 0.1087 (*pec1pec2-1*, -150 mV), *p* = 0.0745 (*pec1pec2-1*, +150 mV), *p* = 0.0595 (*pec1pec2-2*, -150 mV), *p* = 0.0889 (*pec1pec2-2*, +150 mV). (E) Experimental setup for determining PEC cation selectivity: Patch-clamp recordings of isolated chloroplasts were performed in symmetric 250 mM potassium gluconate. (F) Substituting KCl for potassium gluconate does not inhibit envelope currents in Arabidopsis WT and *pec1pec2* chloroplasts. Representative I/V curves are shown, and quantified current amplitudes at -150 mV and +150 mV are plotted (n ≥ 3). Bars represent mean ± SEM; letters indicate statistical significance (one-way ANOVA with Tukey's post-hoc test, $p < 0.05$).

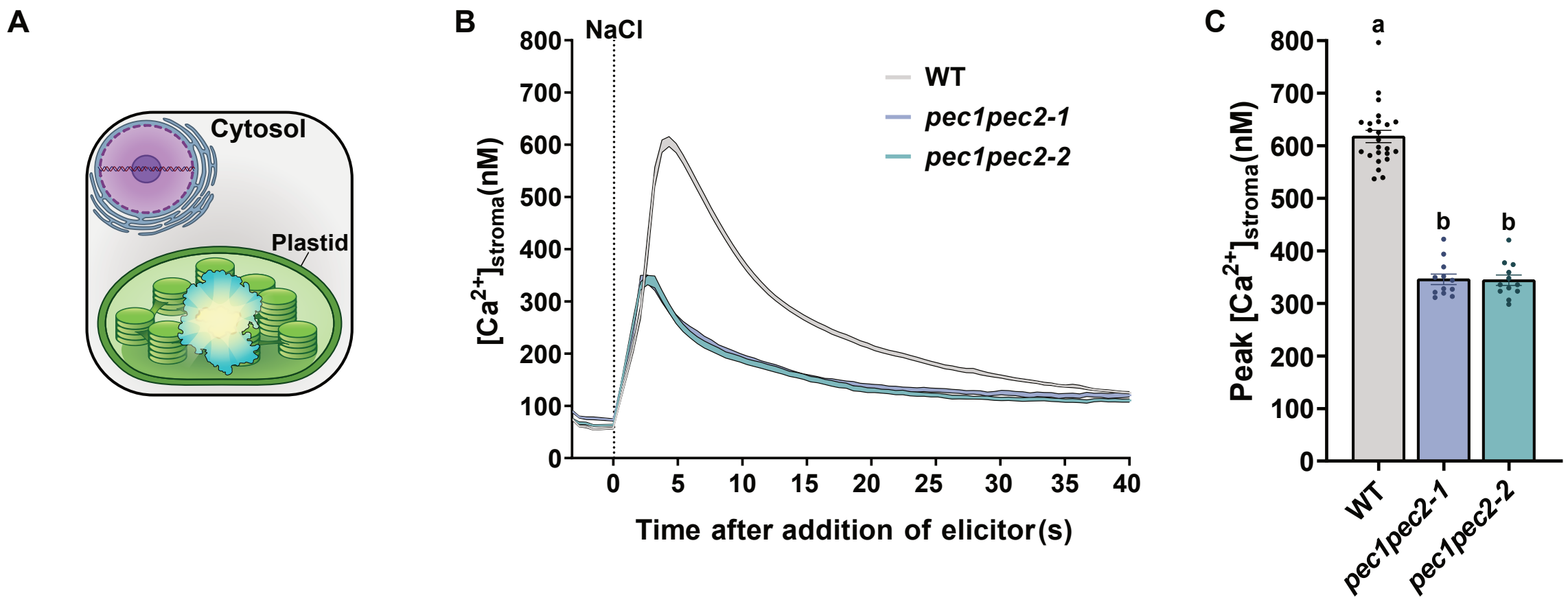

Figure S2: Stromal Ca^{2+} transients are dampened in response to 200 mM NaCl in WT and both *pec1pec2* mutants. (A) Schematic representation of the subcellular localization of the stromal-targeted Aequorin. (B) Stromal Ca^{2+} transients measured in WT and *pec1pec2* seedlings following elicitation with 200 mM NaCl. Lines indicate mean, shaded areas indicate SEM ($n \geq 12$). (C) Quantification of peak stromal Ca^{2+} levels. Bars indicate mean ± SEM ($n \geq 12$). Letters indicate statistical significance (one-way ANOVA with Tukey's test, $p < 0.05$).

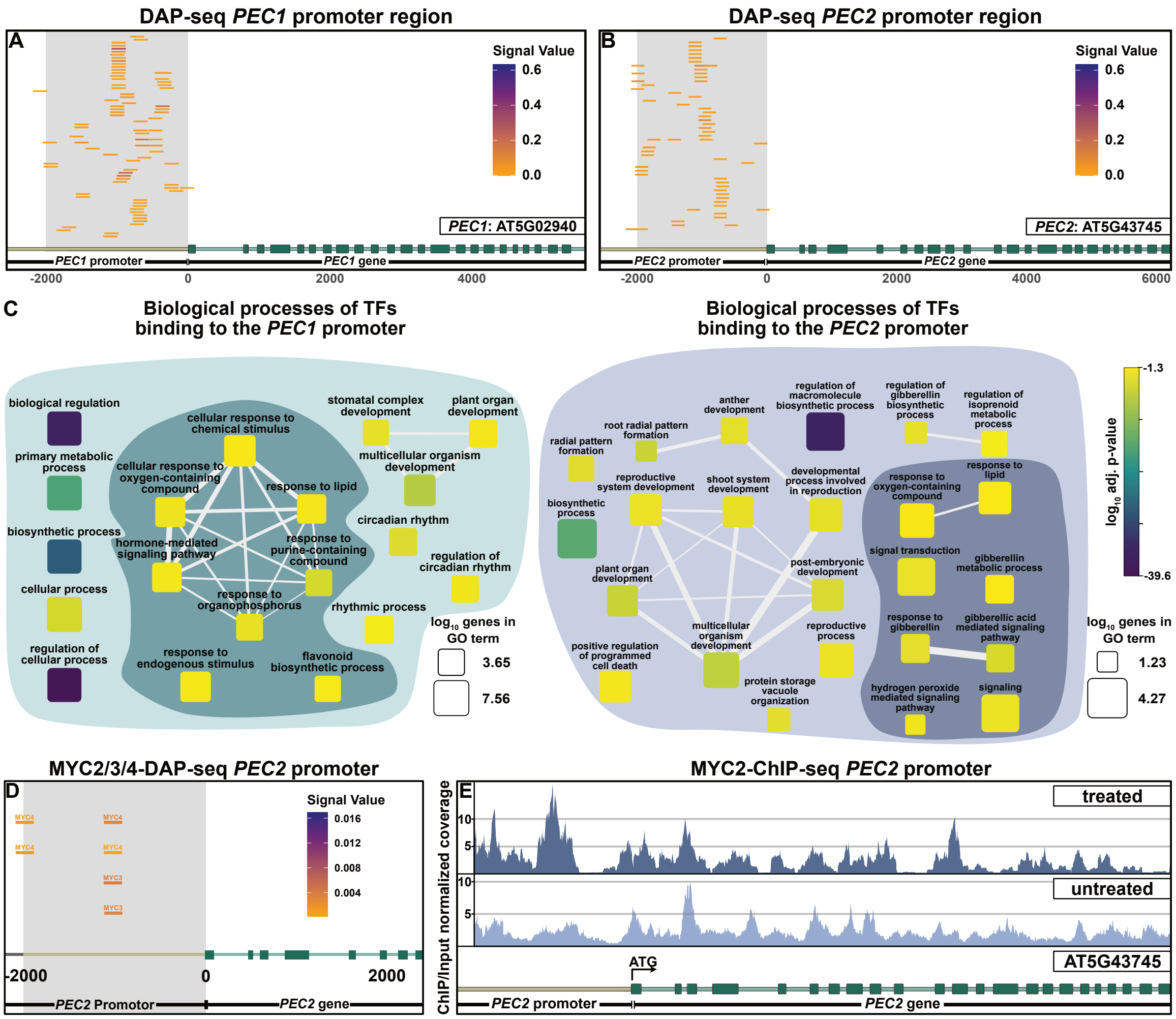

Figure S3: Transcriptional control of PECs indicates stress-dependent regulation. (A, B) DAP-seq profiles showing all TF binding events in the promoter regions (2 kb upstream) of *PEC1* (A) and *PEC2* (B), in two replicates. Binding events are colored from orange to purple by normalized peak height. (C) Selected GO terms from GO term enrichment of TFs binding to the *PEC1* (left) and *PEC2* (right) promoter (g:SCS-corrected, $p < 0.05$). GO terms related to DNA binding were excluded; node size reflects number of genes included in the GO term, color represents $-\log_{10}(p\text{-value})$. GO terms associated with stress/hormone signaling are highlighted in a darker shade. (D) DAP-seq binding profile shows MYC3/4 binding to the *PEC2* promoter. Binding events are colored from orange to purple by normalized peak height. (E) ChIP-seq of MYC2 binding to the *PEC2* promoter in untreated and water-spray treated plants. Enrichment over Input was calculated (bin size = 1 bp), normalized by read count, and plotted over the *PEC2* locus (Chr5:17569000-17580000).

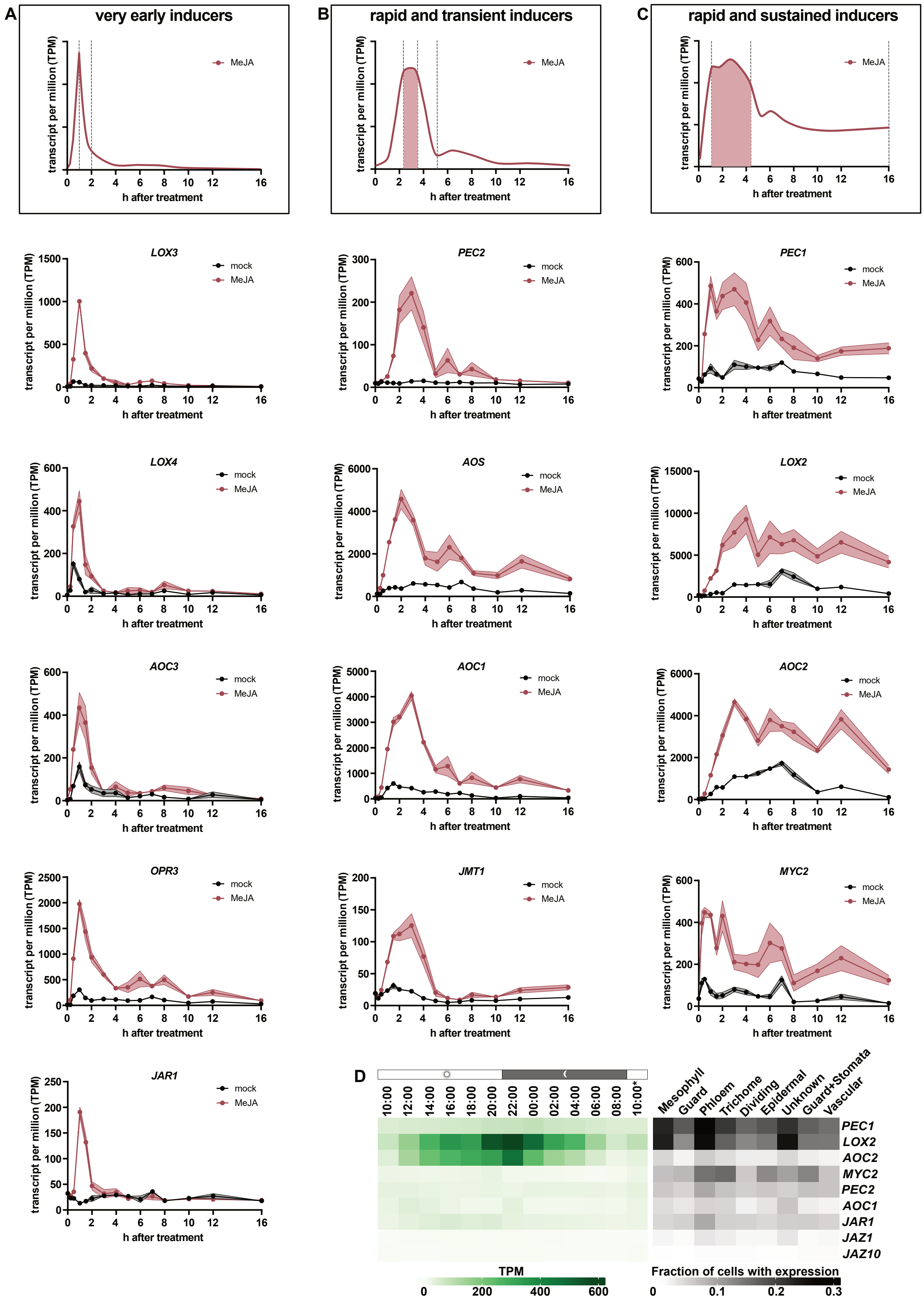

A
very early inducers
B
rapid and transient inducers
C
rapid and sustained inducers
MeJA
mock
transcript per million (TPM)
h after treatment
LOX3
PEC2
PEC1
LOX4
AOS
LOX2
AOC3
AOC1
AOC2
OPR3
JMT1
MYC2
JAR1
D
10:00
12:00
14:00
16:00
18:00
20:00
22:00
00:00
02:00
04:00
06:00
08:00
10:00*
Mesophyll
Guard
Phloem
Trichome
Dividing
Epidermal
Unknown
Guard+Stomata
Vascular
PEC1
LOX2
AOC2
MYC2
PEC2
AOC1
JAR1
JAZ1
JAZ10
TPM
Fraction of cells with expression

Figure S4: PECs are rapidly induced in the same tissues as JA markers upon treatment with MeJA. Re-analysis of time-course RNA-seq from MeJA-treated Arabidopsis (25) was used to group JA-responsive genes into three categories based on their temporal expression profiles, plotted by TPM. A schematic overview of each group's expression pattern is shown at the top of each column. (A) Very early inducers, characterized by a sharp transient peak within the first 1-2 h after MeJA treatment (e.g., *LOX3*, *LOX4*, *AOC3*, *OPR3*, *JAR1*). (B) Rapid and transient inducers, showing peak expression within 1-4 h followed by return to baseline (e.g., *PEC2*, *AOS*, *AOC1*, *JMT1*). (C) Rapid and sustained inducers, showing rapid induction within 1-4 h with sustained expression over 16 h (e.g., *PEC1*, *LOX2*, *AOC2*, *MYC2*). Transcript per million (TPM) is shown for treated and mock samples; shaded areas indicate SEM (n = 4). (D) Heatmap showing diurnal expression (TPM) of selected transcripts for JA marker genes and the fraction of cells with expression of the respective genes for each cell type.

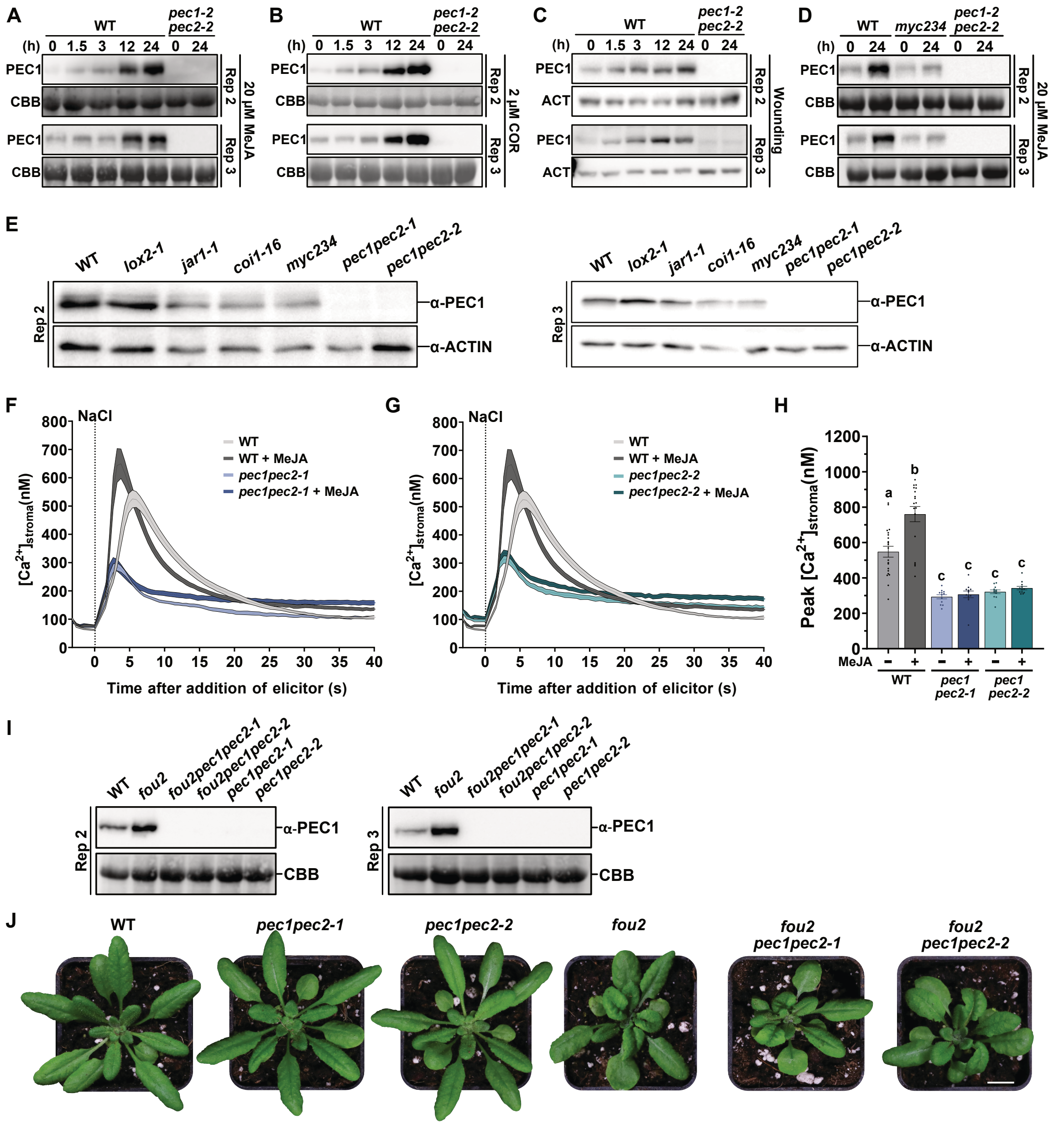

Figure S5: PECs are crucial for MeJA-induced augmented Ca^{2+} transients in WT and *fou2*. (A) Two additional replicates for Fig. 2 *D*: Immunoblot analyses showing PEC1 protein accumulation in 2-week old WT and *pec1pec2-2* at indicated time points following treatment with 20 µM MeJA. Coomassie Brilliant Blue (CBB) staining of RuBisCO LSU serves as loading control. (B) Two additional replicates for Fig. 2 *D*: Immunoblot analyses showing PEC1 protein accumulation in 2-week old WT and *pec1pec2-2* at indicated time points following treatment with 2 µM COR. CBB staining of RuBisCO LSU serves as loading control. (C) Two additional replicates for Fig. 2 *D*: Immunoblot analyses showing PEC1 protein accumulation in 2-week old WT and *pec1pec2-2* at indicated time points following treatment by wounding. ACTIN serves as loading control. (D) Two additional replicates for Fig. 2 *E*: Immunoblot displaying PEC1 levels in 2-week old *myc234* loss-of-function mutants compared to WT and *pec1pec2-2* before and after 24 h of MeJA treatment. CBB staining of RuBisCO functions as loading control. (E) Two additional replicates for Fig. 2 *F*: Basal PEC1 protein levels in 3-week old JA signaling mutants (*lox2-1*, *jar1-1*, *coi1-16*, *myc234*) compared to WT. ACTIN serves as loading control. (F-G) Stromal Ca^{2+} transients after eliciting with 200 mM NaCl in WT, *pec1pec2-1* (F), and *pec1pec2-2* (G) seedlings

pretreated with 200 μM MeJA for 24 h ($n \geq 12$). WT plants treated with MeJA show elevated stromal Ca^{2+} peaks. This effect is absent in both *pec1pec2* mutants. Shaded areas indicate SEM, ($n \geq 12$). (H) Quantification of peak stromal Ca^{2+} levels from (F, G). Bars are mean ± SEM ($n \geq 12$). Letters indicate statistical groups as determined by one-way ANOVA with Tukey's correction. (**I**) Two additional replicates for Fig. 3 *E*: Immunoblot showing basal PEC1 protein levels in WT, *fou2*, *fou2pec1pec2*, and *pec1pec2* mutants. RuBisCO LSU was stained with CBB as loading control. (J) Rosettes of 4-week-old WT, *pec1pec2-1*, *pec1pec2-2*, *fou2*, *fou2pec1pec2-1*, and *fou2pec1pec2-2* plants grown under 16 h light / 8 h dark photoperiod. Scale bar equals 1 cm.

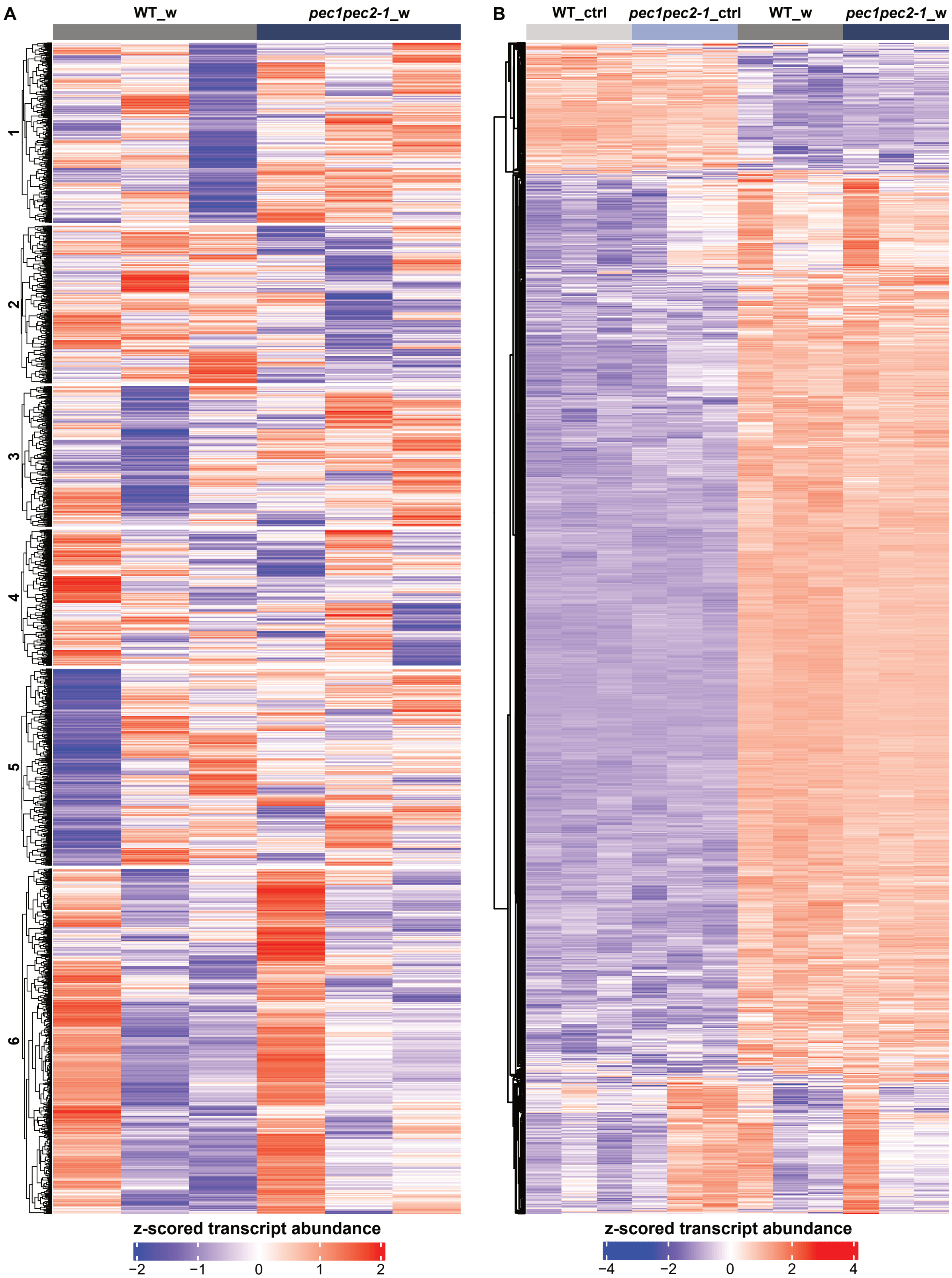

Figure S6: The global wounding response is mostly intact in *pec1pec2* mutants. (A) Heatmap showing k-means clustering (k=6) of *z*-scored transcript abundance (*z*-scores of normalized read counts) of the top 2000 changed genes in the wounding samples by standard deviation in WT (grey) and *pec1pec2-1* (blue) after wounding (n = 3). (B) Heatmap showing Pearson clustering of *z*-scores

of normalized read counts of the top 2000 changed genes in all samples by standard deviation in WT (grey) and *pec1pec2-1* (blue), before (light color) and 90 min after wounding (dark color) (n = 3).

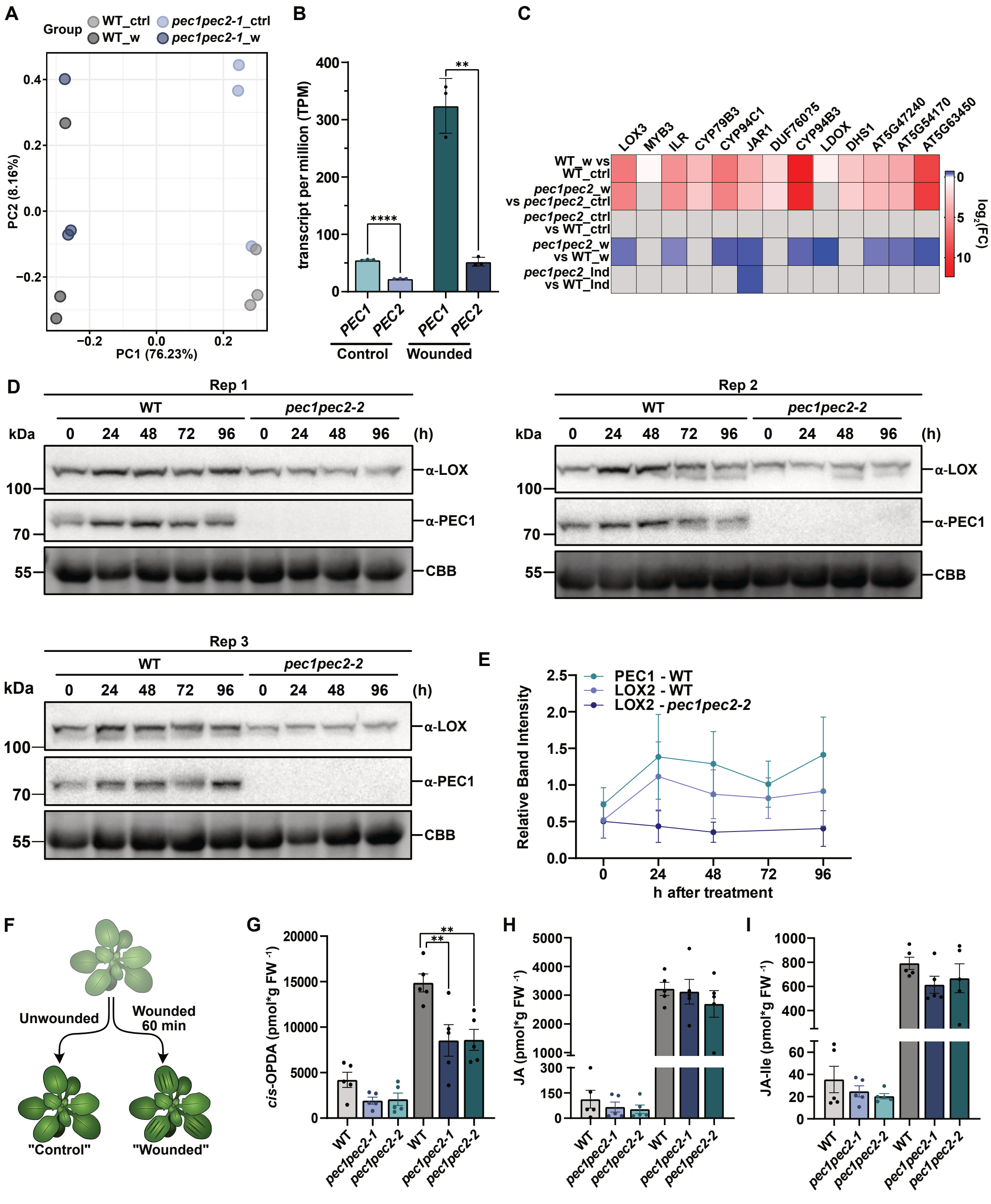

Figure S7: Loss of PECs attenuates induction of a subset of wounding-induced genes but is not detrimental for the global wounding response. (A) Principal component analysis (PCA) of normalized read counts from all RNA-Seq samples. Samples cluster by treatment (control vs wounded) along PC1 (PC1: 76.2%, PC2: 8.2%). (B) Transcript levels (transcripts per million, TPM) of *PEC1* and *PEC2* in WT under control and wounded conditions. Bars represent mean ± SEM (n = 3). Statistical significance was determined by unpaired Welch's t test within conditions (***p* < 0.01; *****p* < 0.0001). (C) Selected DEGs included in the enriched GO-terms "response to wounding" and "response to jasmonic acid" (Benjamini-Hochberg FDR, $p < 0.05$). Induction ((*pec1pec2*_w vs *pec1pec2*_ctrl) vs (WT_w vs WT_ctrl)) abbreviated as Ind. Colors indicate $\log_2$

fold changes of the genes in indicated contrasts. Grey indicates not significantly changed genes. (D) Three replicates of immunoblot analysis of PEC1 and LOX2 protein levels in WT and *pec1pec2-2* plants over 96 h after wounding. RuBisCO LSU stained with CBB serves as loading control. (E) Quantification of band intensities of immunoblots from (D), normalized to RuBisCO LSU. Plot represents mean ± SEM (n = 3) (F) Experimental workflow of the hormone quantification in unprimed plants. WT and *pec1pec2-1* seedlings were wounded or left untreated (Control). Samples were harvested 60 min after wounding. (G-I) Baseline levels of *cis*-OPDA (G), JA (H), and JA-Ile (I) in unprimed WT and *pec1pec2* mutants, before and 60 min after wounding. Bars represent mean ± SEM (n = 5). Statistical significance was assessed within each treatment using two-way ANOVA with Tukey's correction; $^{**}p < 0.01$.

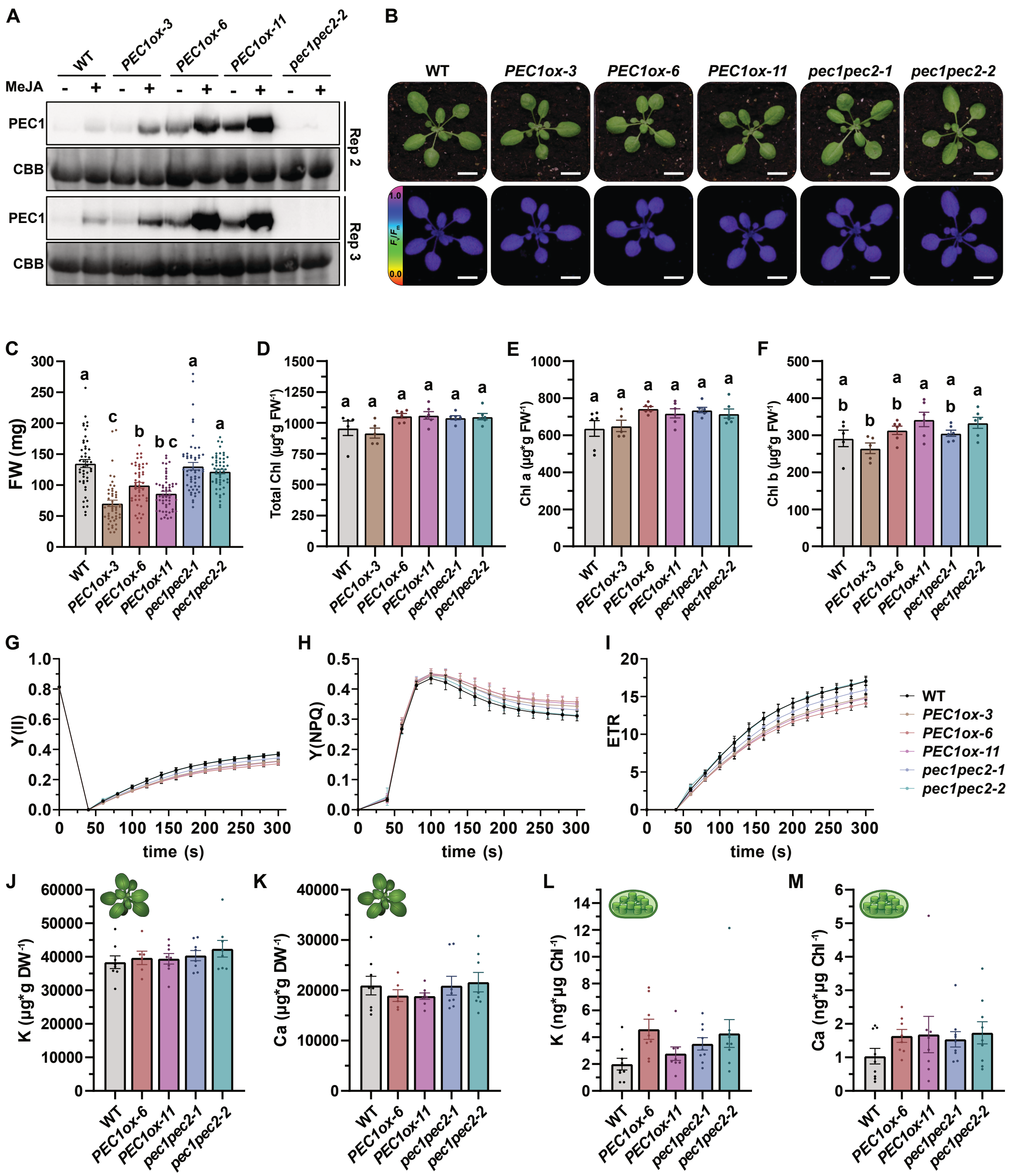

Figure S8: *PEC1* overexpression leads to reduced growth without altering the plastid ionome. (A) Two additional replicates for Fig. 5*B*: Immunoblot confirming JA-responsiveness of the *pPEC1::PEC1* construct. Two-week-old plants were treated with 20 µM MeJA for 24 h. CBB staining of RuBisCO indicates equal loading. (B) RGB and false-color panel showing F_v/F_m of three-week-old WT, *pec1pec2*, and *PEC1ox* lines. Scale bar indicates 1 cm. (C) Fresh weight (FW) of four-week-old plants grown in short-day conditions (12 h light/12 h dark). Bars indicate mean ± SEM (n ≥ 50). Letters indicate statistically distinct groups (one-way ANOVA with Tukey's post-hoc test, $p < 0.05$) (D-F) Total Chlorophyll (D), Chlorophyll a (E), and Chlorophyll b (F) content in three-week-old plants normalized to fresh weight. Bars indicate mean ± SEM (n ≥ 5). Letters indicate statistical significance (one-way ANOVA with Tukey's post-hoc test, $p < 0.05$). (G-I) Photosynthetic parameters measured in three-week-old plants via Imaging-PAM. Graphs show Y(II) (G), Y(NPQ)

(H), and Electron Transport Rate (ETR) (I) during a standard 5-min light curve at 110 μmol photons m^{-2} s^{-1} (n = 6). (J, K) Whole rosette ion levels of potassium (J) and calcium (K) in three-week old plants normalized to dry weight (DW). Bars indicate mean ± SEM (n ≥ 6). Letters denote statistically distinct groups (one-way ANOVA with Tukey's post-hoc test, $p < 0.05$). (L, M) Potassium (L) and calcium (M) content in isolated chloroplasts normalized to chlorophyll content. Bars indicate mean ± SEM (n ≥ 6). Letters denote statistically distinct groups (one-way ANOVA with Tukey's post-hoc test, $p < 0.05$).

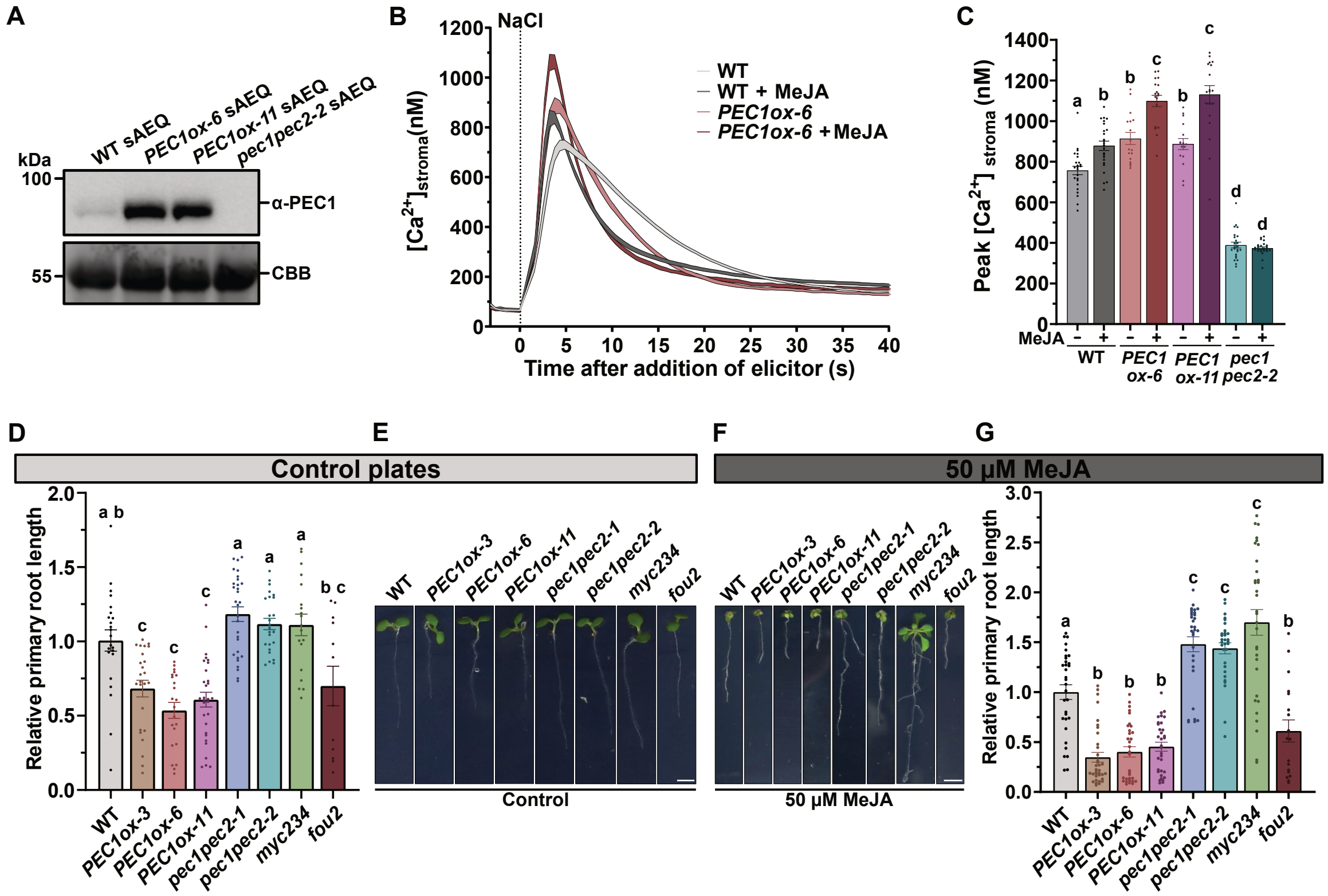

Figure S9: Stromal Ca^{2+} transients as well as sensitivity to MeJA are exacerbated in *PEC1ox* mutant lines. (A) Immunoblot showing PEC1 protein levels in three-week old plants expressing plastid-targeted YFP-Aequorin (sAEQ) in WT, *pec1pec2-2* and *PEC1*ox-6/11 background. CBB staining of RuBisCO LSU serves as loading control. (B) Stromal Ca^{2+} transients measured in WT and *PEC1ox-6* seedlings with or without treatment with 200 µM MeJA following elicitation with 200 mM NaCl. Lines indicate mean, shaded areas indicate SEM (n ≥ 17). (C) Quantification of peak stromal Ca^{2+} concentrations shown in (B) and *PEC1ox-11* and *pec1pec2-2* of the same measurement. Bars represent mean ± SEM (n ≥ 16). Letters denote statistically distinct groups (one-way ANOVA with Tukey's post-hoc test, $p < 0.05$). (D) Primary root length of untreated, 5-day old seedlings grown vertically on ½ MS medium. Bars represent mean ± SEM (n ≥ 15; n = 5 for *fou2*). Letters represent statistically distinct groups (one-way ANOVA with Tukey's post-hoc test, $p < 0.05$). (E) Representative images of the root length of 5-day old seedlings under control conditions on ½ MS plates. (F) Representative images of the root length of seedlings transferred to ½ MS plates supplemented with 50 µM MeJA at 4 days old for 7 days. Images are representative of three biological replicates. (G) Quantification of the primary root length of the seedlings grown on ½ MS plates supplemented with 50 µM MeJA shown in (F). Bars represent mean ± SEM (n ≥ 15; n = 5 for *fou2*). Letters represent statistically distinct groups (one-way ANOVA with Tukey's post-hoc test, $p < 0.05$).

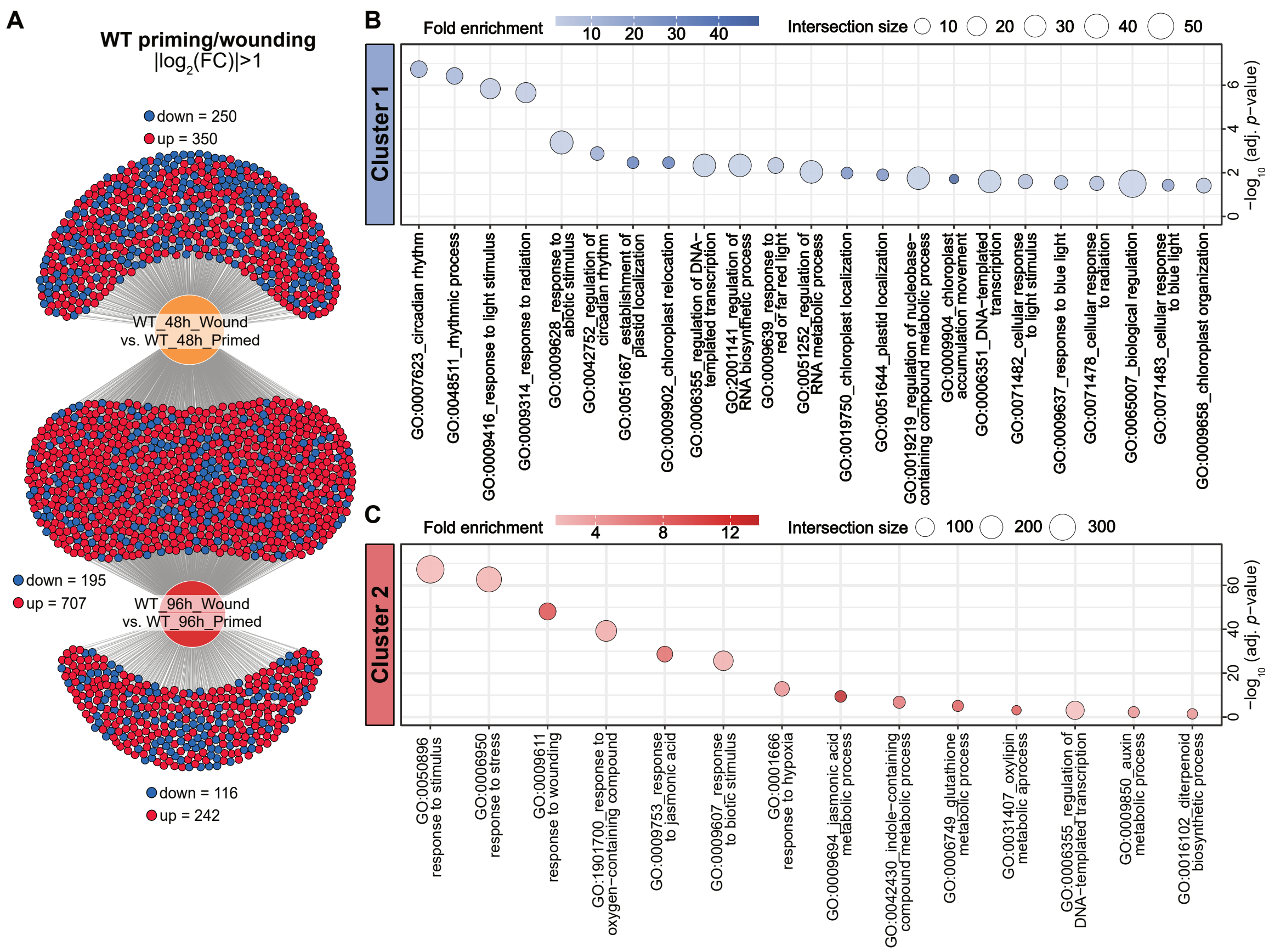

Figure S10: Repeated wounding induces a JA-dependent priming response in WT. (A) DiVenn diagram showing the overlap of differentially expressed genes in WT for 48 h_Wound vs 48 h_Primed and 96 h_Wound vs 96 h_Primed. Nodes represent individual genes, with blue and red indicating downregulated and upregulated genes, respectively. (B) Selected enriched GO-terms of DEGs in Cluster 1 (g:SCS corrected $p < 0.05$) (Fig. 5 *F*). Bubble size corresponds to the intersection size (number of DEGs annotated in the GO term), and coloring depicts fold enrichment. (C) Selected enriched GO terms of DEGs in Cluster 2 (g:SCS corrected, $p < 0.05$) (Fig. 5 *F*). Bubble size corresponds to the intersection size (number of DEGs annotated in the GO term), and coloring depicts fold enrichment.

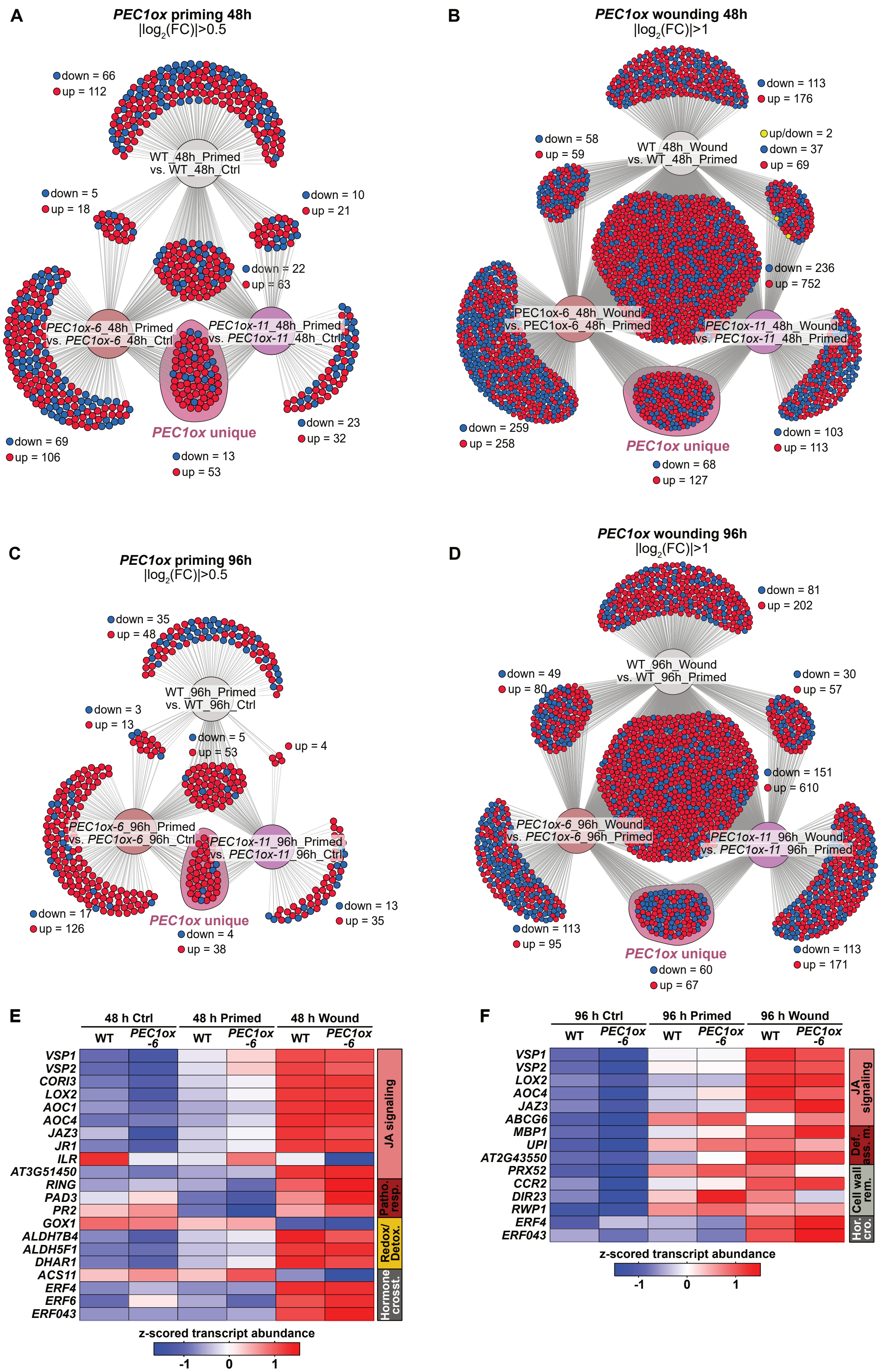

A
PEC1ox priming 48h
|log2(FC)|>0.5
down = 66
up = 112
WT_48h_Primed vs. WT_48h_Ctrl
down = 5
up = 18
down = 10
up = 21
down = 22
up = 63
PEC1ox-6_48h_Primed vs. PEC1ox-6_48h_Ctrl
PEC1ox-11_48h_Primed vs. PEC1ox-11_48h_Ctrl
PEC1ox unique
down = 69
up = 106
down = 13
up = 53
down = 23
up = 32
B
PEC1ox wounding 48h
|log2(FC)|>1
down = 113
up = 176
down = 58
up = 59
WT_48h_Wound vs. WT_48h_Primed
up/down = 2
down = 37
up = 69
down = 236
up = 752
PEC1ox-6_48h_Wound vs. PEC1ox-6_48h_Primed
PEC1ox-11_48h_Wound vs. PEC1ox-11_48h_Primed
down = 259
up = 258
PEC1ox unique
down = 68
up = 127
down = 103
up = 113
C
PEC1ox priming 96h
|log2(FC)|>0.5
down = 35
up = 48
WT_96h_Primed vs. WT_96h_Ctrl
down = 3
up = 13
down = 5
up = 53
up = 4
PEC1ox-6_96h_Primed vs. PEC1ox-6_96h_Ctrl
PEC1ox-11_96h_Primed vs. PEC1ox-11_96h_Ctrl
down = 17
up = 126
PEC1ox unique
down = 4
up = 38
down = 13
up = 35
D
PEC1ox wounding 96h
|log2(FC)|>1
down = 81
up = 202
down = 49
up = 80
WT_96h_Wound vs. WT_96h_Primed
down = 30
up = 57
down = 151
up = 610
PEC1ox-6_96h_Wound vs. PEC1ox-6_96h_Primed
PEC1ox-11_96h_Wound vs. PEC1ox-11_96h_Primed
down = 113
up = 95
PEC1ox unique
down = 60
up = 67
down = 113
up = 171
E
48 h Ctrl
48 h Primed
48 h Wound
WT
PEC1ox -6
VSP1
VSP2
CORI3
LOX2
AOC1
AOC4
JAZ3
JR1
ILR
AT3G51450
RING
PAD3
PR2
GOX1
ALDH7B4
ALDH5F1
DHAR1
ACS11
ERF4
ERF6
ERF043
JA signaling
Patho. resp.
Redox/ Detox.
Hormone crosst.
z-scored transcript abundance
-1
0
1
F
96 h Ctrl
96 h Primed
96 h Wound
WT
PEC1ox -6
VSP1
VSP2
LOX2
AOC4
JAZ3
ABCG6
MBP1
UPI
AT2G43550
PRX52
CCR2
DIR23
RWP1
ERF4
ERF043
JA signaling
Def. ass. m.
Cell wall rem.
Hor. cro.
z-scored transcript abundance
-1
0
1

Figure S11: A subset of genes is uniquely induced in *PEC1ox* upon priming and wounding. (A-D) DiVenn diagrams showing the overlap of genes differentially expressed in 48 h_Primed vs 48 h_Control (A), 48 h_Wound vs 48 h_Primed (B), 96 h_Primed vs 96 h_Control (C), and 96 h_Wound vs 96 h_Primed (D) comparisons for WT, *PEC1ox-6*, and *PEC1ox-11*. Nodes represent individual genes, with blue and red indicating downregulated and upregulated genes, respectively. (E) Heatmap showing *z*-scored transcript abundance (*z*-scores of normalized read counts) of selected genes shown in Fig. 5 *I* in the 48 h Control, 48 h Primed, and 48 h Wound samples in WT and *PEC1ox-6* (n = 3). Genes were grouped by functional categories: JA signaling, pathogen response, redox/detoxification, and hormone crosstalk. (F) Heatmap showing *z*-scored transcript abundance (*z*-scores of normalized read counts) of selected genes shown in Fig. 5 *J* in the 96 h Control, 96 h Primed, and 96 h Wound samples in WT and *PEC1ox-6* (n = 3). Selected genes are grouped by functional categories: JA signaling, defense associated molecules, cell wall remodeling, and hormone crosstalk. Genes shown were significantly differentially expressed in *PEC1ox* compared to WT in at least one comparison across all priming and wounding conditions.

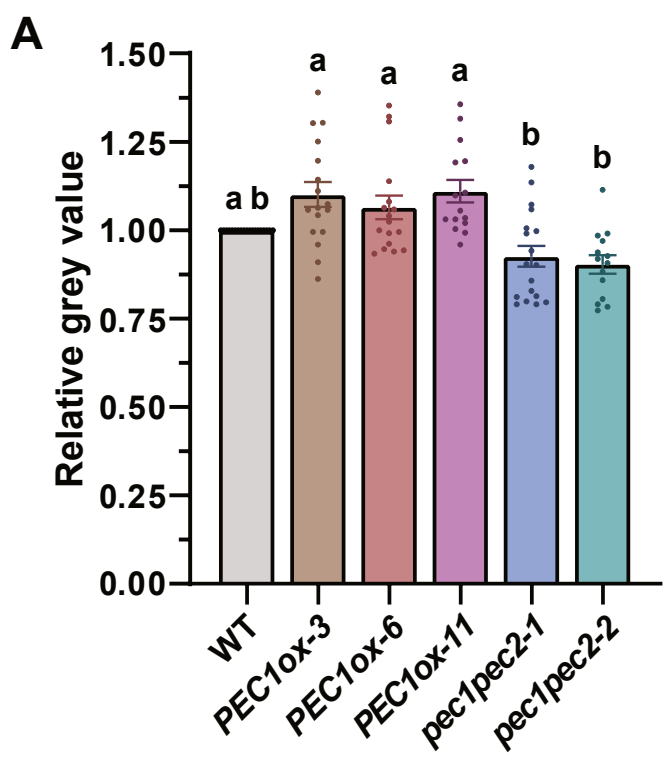

Figure S12: *PEC1* overexpression increases resistance against *Botrytis cinerea*. (A) Quantification of the intensity of all Trypan Blue stainings in leaves infected with *Botrytis cinerea* (2 dpi) using Fiji, normalized to WT. Images were converted to greyscale and the mean grey value was quantified within a consistent ROI. Higher values indicate more intense staining and thus larger primary lesions. Bars represent mean ± SEM (n ≥ 14). Letters represent statistically distinct groups (one-way ANOVA with Tukey's post-hoc test, $p < 0.05$).

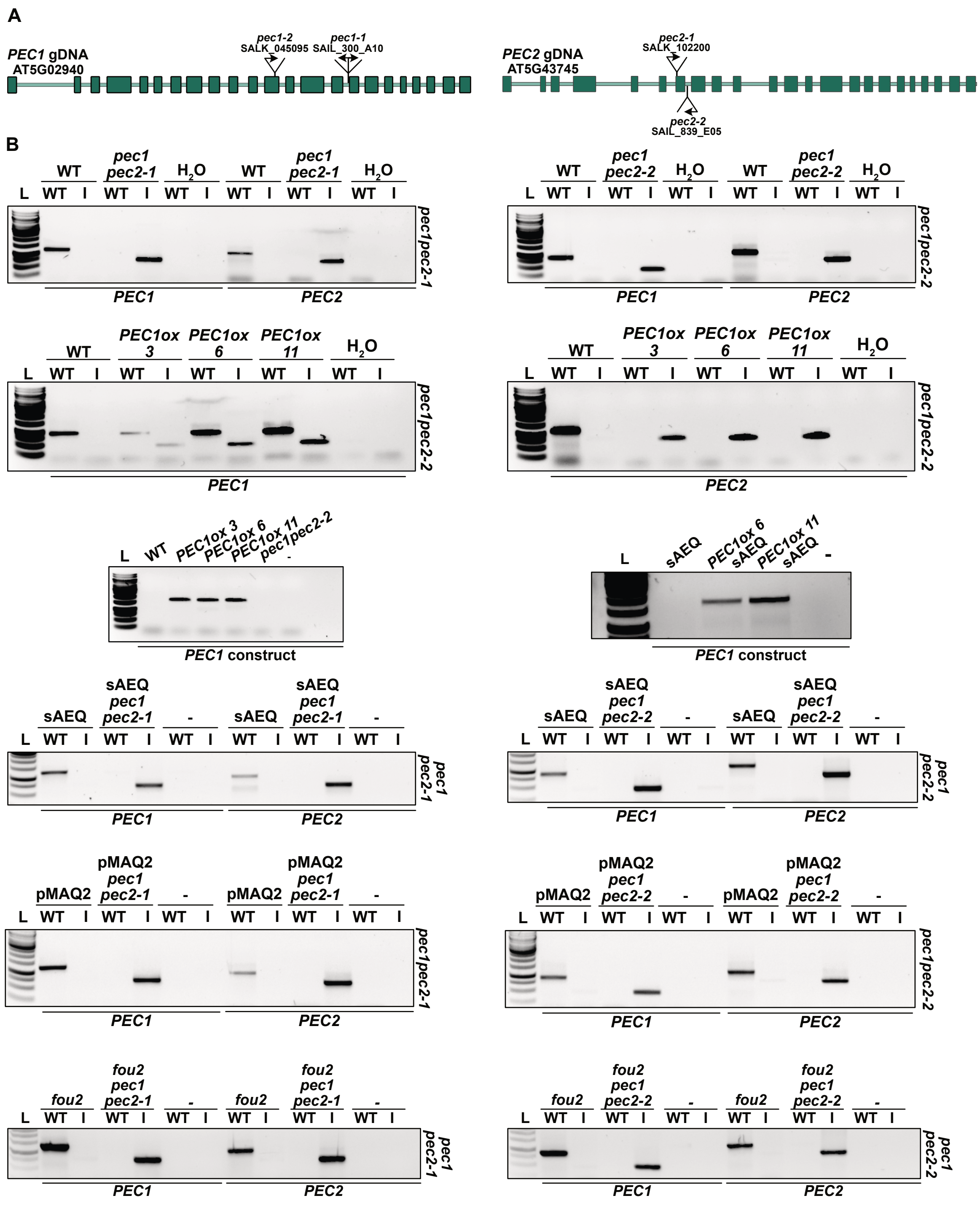

Figure S13: Genotypic analysis of all newly generated mutant lines. (A) Annotations of T-DNA insertion lines used in this study. *PEC1* (AT5G02940) (left) and *PEC2* (AT5G43745) (right) gene models show sites of T-DNA insertion and binding sites of the genotyping primers. Exons are depicted as green boxes. (B) Confirmation of higher order mutants by PCR. WT and I indicate the usage of primer combinations specific for the wild-type and T-DNA insertion alleles, respectively.

Dataset S1 (separate file). TF binding analysis of *PEC* promoters via DAP-Seq. (A) All TFs binding to the *PEC1* promoter from two replicates (indicated in column "method"). (B) All TFs binding to the *PEC2* promoter from two replicates (indicated in column "method"). Signal values were normalized within each TF experiment

Dataset S2 (separate file). GO term enrichment analysis of TFs binding to *PEC* promoters. (A) Selected GOs enriched for the *PEC1* promoter (g:SCS corrected, p < 0.05). (B) Selected GOs enriched for the *PEC2* promoter (g:SCS corrected, p < 0.05).

Dataset S3 (separate file). Gene list obtained by exploratory k-means clustering (k=6) of the top 2000 most variable genes 90 min after wounding of WT and *pec1pec2-1* for the RNA-Seq dataset 1. Includes all 6 clusters.

Dataset S4 (separate file). GO term enrichment analysis of genes in wounding cluster_2 for RNA-Seq dataset 1 (Benjamini-Hochberg, FDR < 0.05).

Dataset S5 (separate file). DEGs with corresponding log_2FC and adj. p value identified by RNA-Seq in *pec1pec2-1* vs WT before (ctrl) and after wounding (w) and differences of transcriptional wounding responses for the RNA-Seq dataset 1.

Dataset S6 (separate file). GO term enrichment analysis of DEGs obtained from RNA-Seq dataset 1 (Benjamini-Hochberg, FDR < 0.05).

Dataset S7 (separate file). Ionome analysis in WT, *pec1pec2*, and *PEC1ox* mutants. (A) Metal ion analysis of leaf tissue of whole rosettes, in µg*g DW^{-1}. (B) Metal ion analysis of isolated chloroplasts, in ng*µg Chl^{-1}.

Dataset S8 (separate file). Limma-voom calculated DEGs with corresponding log_2FC and adj. p values identified by RNA-Seq of WT, *PEC1ox-6* and *PEC1ox-11* plants at different time points. Contrasts are grouped into 48 h primed (A), 96 h primed (B), 48 h wound (C) and 96 h wound (D) tabs.

Dataset S9 (separate file). GO term enrichment analysis of DEGs obtained from RNA-Seq dataset 2 (g:SCS corrected, p < 0.05). GO term enrichment of cluster 1 (A) and cluster 2 (B) DEGs in WT at 48 h and 96 h primed and wound. GO term enrichment of DEGs uniquely responsive in *PEC1ox-6/-11* at 48 h primed (C), 96 h primed (D), 48 h wound (E), and 96 h wound (F).

Dataset S10 (separate file). Primer combinations used for mutant generation and genotyping. (A) Primer combinations for genotyping. (B) Primer combination for cloning.

SI References

1. T. Murashige, F. Skoog, A Revised Medium for Rapid Growth and Bio Assays with Tobacco Tissue Cultures. *Physiol Plant.* **15**, 473–497 (1962).
2. S. Schwartz *et al.*, Catch & Release—rapid cost-effective protein purification from plants using a DIY GFP-Trap-protease approach. *Plant J.* **124**, e70544 (2025).
3. S. J. Clough, A. F. Bent, Floral dip: a simplified method for Agrobacterium-mediated transformation of Arabidopsis thaliana. *Plant J.* **16**, 735–743 (1998).
4. R. P. Hellens, E. A. Edwards, N. R. Leyland, S. Bean, P. M. Mullineaux, pGreen: a versatile and flexible binary Ti vector for Agrobacterium-mediated plant transformation. *Plant Mol Biol* **42**, 819–832 (2000).
5. H. Aronsson, P. Jarvis, A simple method for isolating import-competent Arabidopsis chloroplasts. *FEBS Lett* **529**, 215–220 (2002).
6. D. I. Arnon, Copper Enzymes in isolated Chloroplasts. Polyphenoloxidase in *Beta vulgaris*. *Plant Physiol.* **24**, 1–15 (1949).
7. I. I. Pottosin, J. Muñiz, S. Shabala, Fast-activating Channel Controls Cation Fluxes across the Native Chloroplast Envelope. *J. Membr. Biol.* **204**, 145–156 (2005).
8. M. R. Knight, A. K. Campbell, S. M. Smith, A. J. Trewavas, Transgenic plant aequorin reports the effects of touch and cold-shock and elicitors on cytoplasmic calcium. *Nature* **352**, 524–526 (1991).
9. C. Völkner *et al.*, Two plastid POLLUX ion channel-like proteins are required for stress-triggered stromal Ca2+ release. *Plant Physiol* **187**, 2110–2125 (2021).
10. N. Mehlmer *et al.*, A toolset of aequorin expression vectors for in planta studies of subcellular calcium concentrations in Arabidopsis thaliana. *J Exp Bot.* **63**, 1751–1761 (2012).
11. K. Tanaka, J. Choi, G. Stacey, "Aequorin Luminescence-Based Functional Calcium Assay for Heterotrimeric G-Proteins in Arabidopsis" in G Protein-Coupled Receptor Signaling in Plants: Methods and Protocols, M. P. Running, Ed. (2013), vol. 1043, pp. 45–54.
12. H. Knight, A. J. Trewavas, M. R. Knight, Cold calcium signaling in Arabidopsis involves two cellular pools and a change in calcium signature after acclimation. *Plant Cell* **8**, 489–503 (1996).
13. C. Völkner *et al.*, Evidence for partial functional overlap of KEA and MSL transport proteins in the chloroplast inner envelope of Arabidopsis thaliana. *FEBS Lett* **598**, 1877–1887 (2024).
14. J. Schindelin *et al.*, Fiji: an open-source platform for biological-image analysis. *Nature Methods* **9**, 676–682 (2012).
15. I. López-Vidriero *et al.*, DNA features beyond the transcription factor binding site specify target recognition by plant MYC2-related bHLH proteins. *Plant Commun* **2**, 100232 (2021).
16. R. C. O'Malley *et al.*, Cistrome and Epicistrome Features Shape the Regulatory DNA Landscape. *Cell* **165**, 1280–1292 (2016).
17. L. Kolberg *et al.*, g:Profiler-interoperable web service for functional enrichment analysis and gene identifier mapping (2023 update). *Nucleic Acids Res* **51**, W207–w212 (2023).
18. F. Supek, M. Bošnjak, N. Škunca, T. Šmuc, REVIGO Summarizes and Visualizes Long Lists of Gene Ontology Terms. *PLOS ONE* **6**, e21800 (2011).
19. P. Shannon *et al.*, Cytoscape: a software environment for integrated models of biomolecular interaction networks. *Genome Res* **13**, 2498–2504 (2003).
20. A. Van Moerkercke *et al.*, A MYC2/MYC3/MYC4-dependent transcription factor network regulates water spray-responsive gene expression and jasmonate levels. *Proc Natl Acad Sci U S A.* **116**, 23345–23356 (2019).
21. C. The Galaxy, The Galaxy platform for accessible, reproducible and collaborative biomedical analyses: 2022 update. *Nucleic Acids Research* **50**, W345–W351 (2022).

22. T. Z. Berardini *et al.*, The Arabidopsis Information Resource: Making and Mining the 'Gold Standard' Annotated Reference Plant Genome. *Genesis (New York, N.Y. : 2000)* **53**, 474–485 (2015).
23. H. Li, Aligning sequence reads, clone sequences and assembly contigs with BWA-MEM. *arXiv pre-print server* arxiv:1303.3997 (2013).
24. F. Ramírez *et al.*, deepTools2: a next generation web server for deep-sequencing data analysis. *Nucleic Acids Res* **44**, W160–165 (2016).
25. R. Hickman *et al.*, Architecture and Dynamics of the Jasmonic Acid Gene Regulatory Network. *Plant Cell.* **29**, 2086–2105 (2017).
26. N. L. Bray, H. Pimentel, P. Melsted, L. Pachter, Near-optimal probabilistic RNA-seq quantification. *Nat Biotechnol* **34**, 525–527 (2016).
27. S. Zenker, K. Schiller, A. Bräutigam, Time- and space-resolved transcriptional regulation in Arabidopsis thaliana. *Plant Biology* **n/a** (2026).
28. T. A. Lee *et al.*, A single-cell, spatial transcriptomic atlas of the Arabidopsis life cycle. *Nature Plants* **11**, 1960–1975 (2025).
29. M. Krämer *et al.*, Cyclic electron flow compensates loss of PGDH3 and concomitant stromal NADH reduction. *Sci Rep* **14**, 29274 (2024).
30. A. Dobin *et al.*, STAR: ultrafast universal RNA-seq aligner. *Bioinformatics* **29**, 15–21 (2013).
31. Z. Jiang *et al.*, Deciphering acclimation to sublethal combined and sequential abiotic stresses in Arabidopsis thaliana. *Plant Physiol* **200** (2026).
32. A. Verhoeven *et al.*, Arabidopsis latent virus 1, a comovirus widely spread in Arabidopsis thaliana collections. *New Phytol* **237**, 1146–1153 (2023).
33. L. Wang, S. Wang, W. Li, RSeQC: quality control of RNA-seq experiments. *Bioinformatics* **28**, 2184–2185 (2012).
34. Y. Liao, G. K. Smyth, W. Shi, featureCounts: an efficient general purpose program for assigning sequence reads to genomic features. *Bioinformatics* **30**, 923–930 (2014).
35. C. W. Law, Y. Chen, W. Shi, G. K. Smyth, voom: Precision weights unlock linear model analysis tools for RNA-seq read counts. *Genome Biol* **15**, R29 (2014).
36. M. D. Robinson, D. J. McCarthy, G. K. Smyth, edgeR: a Bioconductor package for differential expression analysis of digital gene expression data. *Bioinformatics* **26**, 139–140 (2010).
37. R. Liu *et al.*, Why weight? Modelling sample and observational level variability improves power in RNA-seq analyses. *Nucleic Acids Res* **43**, e97 (2015).
38. S. X. Ge, E. W. Son, R. Yao, iDEP: an integrated web application for differential expression and pathway analysis of RNA-Seq data. *BMC Bioinformatics* **19**, 534 (2018).
39. S. Tyanova *et al.*, The Perseus computational platform for comprehensive analysis of (prote)omics data. *Nat Methods* **13**, 731–740 (2016).
40. H. Wickham, *ggplot2: Elegant Graphics for Data Analysis* (Springer New York, NY, 2009), https://doi.org/10.1007/978-0-387-98141-3, pp. VIII, 213.
41. P. Bardou, J. Mariette, F. Escudié, C. Djemiel, C. Klopp, jvenn: an interactive Venn diagram viewer. *BMC Bioinformatics* **15**, 293 (2014).
42. L. Sun *et al.*, DiVenn: An Interactive and Integrated Web-Based Visualization Tool for Comparing Gene Lists. *Front Genet* **10**, 421 (2019).
43. H.-H. Kunz *et al.*, The ABC Transporter PXA1 and Peroxisomal β-Oxidation Are Vital for Metabolism in Mature Leaves of Arabidopsis during Extended Darkness. *Plant Cell.* **21**, 2733–2749 (2009).
44. H. K. Lichtenthaler, Chlorophylls and carotenoids: Pigments of photosynthetic biomembranes. *Method Enzymol.* **148**, 350–382 (1987).
45. R. J. Porra, W. A. Thompson, P. E. Kriedemann, Determination of accurate extinction coefficients and simultaneous equations for assaying chlorophylls a and b extracted with four different solvents: verification of the concentration of chlorophyll standards by atomic absorption spectroscopy. *Biochimica et Biophysica Acta (BBA) - Bioenergetics* **975**, 384–394 (1989).

46. R. Höhner, S. Tabatabaei, H.-H. Kunz, U. Fittschen, A rapid total reflection X-ray fluorescence protocol for micro analyses of ion profiles in Arabidopsis thaliana. *Spectrochimica Acta Part B: Atomic Spectroscopy* **125**, 159–167 (2016).
47. L. J. Holzner *et al.*, The chloroplast ionome shines light on the dynamics of organellar iron homeostasis. *Plant Cell.* **38**, koag017 (2026).
48. A. Porquier *et al.*, Retrotransposons as pathogenicity factors of the plant pathogenic fungus Botrytis cinerea. *Genome Biology* **22** (2021).
49. E. Koch, A. Slusarenko, Arabidopsis is susceptible to infection by a downy mildew fungus. *Plant Cell.* **2**, 437–445 (1990).
50. P. E. Staswick, W. Su, S. H. Howell, Methyl jasmonate inhibition of root growth and induction of a leaf protein are decreased in an Arabidopsis thaliana mutant. *Proc Natl Acad Sci U S A.* **89**, 6837–6840 (1992).
51. K. Mekkaoui *et al.*, Transcriptomics and trans-organellar complementation reveal limited signaling of 12-cis-oxo-phytodienoic acid during early wound response in Arabidopsis. *Nature communications* **16** (2025).
52. G. U. Balcke *et al.*, An UPLC-MS/MS method for highly sensitive high-throughput analysis of phytohormones in plant tissues. *Plant Methods* **8**, 47 (2012).
53. G. Bonaventure *et al.*, A gain-of-function allele of TPC1 activates oxylipin biogenesis after leaf wounding in Arabidopsis. *Plant J.* **49**, 889–898 (2007).
54. G. Glauser *et al.*, Velocity estimates for signal propagation leading to systemic jasmonic acid accumulation in wounded Arabidopsis. *J Biol Chem* **284**, 34506–34513 (2009).
55. P. Fernández-Calvo *et al.*, The Arabidopsis bHLH Transcription Factors MYC3 and MYC4 Are Targets of JAZ Repressors and Act Additively with MYC2 in the Activation of Jasmonate Responses. *Plant Cell.* **23**, 701–715 (2011).
56. C. Ellis, J. G. Turner, A conditionally fertile coi1 allele indicates cross-talk between plant hormone signalling pathways in Arabidopsis thaliana seeds and young seedlings. *Planta* **215**, 549–556 (2002).
